# A Role for Polo-Like Kinase 4 in Vascular Fibroblast Cell-Type Transition

**DOI:** 10.1016/j.jacbts.2020.12.015

**Published:** 2021-03-22

**Authors:** Jing Li, Go Urabe, Yitao Huang, Mengxue Zhang, Bowen Wang, Lynn Marcho, Hongtao Shen, K. Craig Kent, Lian-Wang Guo

**Affiliations:** a Department of Surgery, School of Medicine, University of Virginia, Charlottesville, Virginia, USA; b Cellular and Molecular Pathology Graduate Program, School of Medicine and Public Health, University of Wisconsin, Madison, Wisconsin, USA; c Davis Heart and Lung Research Institute, Wexner Medical Center, Ohio State University, Columbus, Ohio, USA

**Keywords:** BRD4, fibroblast cell-type transition, PDGF receptor, PLK4, SRF, αSMA, α-smooth muscle actin, AA, PDGF-AA, BET, bromo/extraterminal domain–containing protein, BRD4, bromodomain protein 4, CenB, centrinone-B, EEL, external elastic lamina, JQ1, a BET family–selective epigenetic modulator drug, MRTF-A, myocardin-related transcription factor A, PDGF, platelet-derived growth factor, PDGFR, PDGF receptor, PLK, polo-like kinase, SRF, serum response factor

## Abstract

Polo-like kinase 4 (PLK4) is canonically known for its cytoplasmic function in centriole duplication. Here we show a noncanonical PLK4 function of regulating the transcription factor SRF’s nuclear activity and associated myofibroblast-like cell-type transition. In this context, we have further found that PLK4’s phosphorylation and transcription are respectively regulated by PDGF receptor and epigenetic factor BRD4. Furthermore, in vivo experiments suggest PLK4 inhibition as a potential approach to mitigating vascular fibrosis.

## Highlights

• PLK4, previously known as a centriole-associated factor, regulates the transcription factor activity of serum response factor.
• PLK4 inhibition blocks the profibrogenic cell state transition of vascular fibroblasts.
• PLK4’s activation and gene expression are regulated by PDGF receptor and epigenetic reader BRD4, respectively.
• Periadventitial administration of a PLK4 inhibitor mitigates vascular fibrosis.

## Visual Abstract

**Figure:**
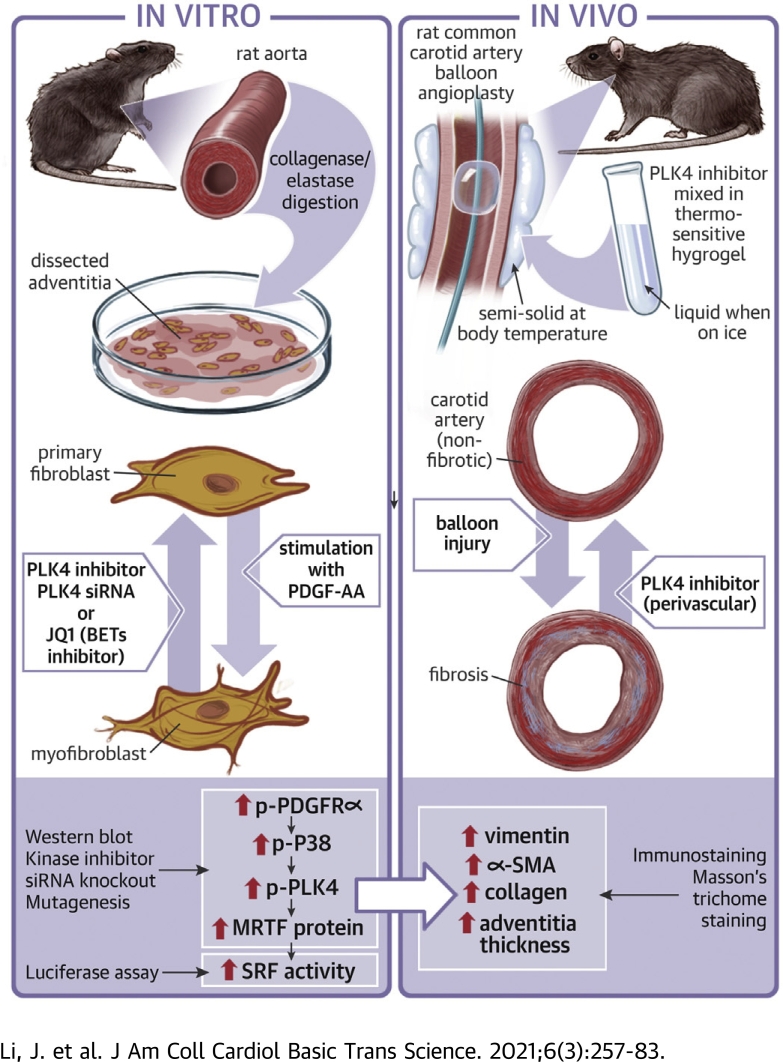

Polo-like kinases (PLKs) regulate cell cycle entry and exit. Among the 5 PLK family members. PLK1 has well documented roles in multiple steps of mitosis. PLK4, the divergent family member with little homology to the other 4 PLKs (1), is reportedly a master regulator of centriole duplication (2), but its noncanonical functions are still obscure (3). Moreover, although a few substrates of PLK4 kinase activity were recently identified (e.g., STIL [SCL/TAL1–interrupting locus] in centriole formation), regulators of PLK4 activation and expression remain poorly defined (3,4). Recent reports implicate PLKs in fibrogenic processes. For example, PLK1 is found to be a target gene of FoxM1, a transcription factor that promotes lung fibrosis (5,6). This raises the question of whether fibroblast cell-type transitions involved in fibrosis are regulated by one or more PLKs.

A well known fibroblast cell-type change is its transition into myofibroblast, a fibrogenic process involved in numerous disease conditions (7). Although the definition of myofibroblasts is still debated, these cells are generally characterized by smooth muscle–like morphologies and proliferative/migratory behaviors (8). Moreover, they often exhibit high levels of α-smooth muscle actin (αSMA), vimentin, platelet-derived growth factor receptor α (PDGFRα), and extracellular matrix proteins (e.g., collagen). Whereas a variety of cell types can differentiate into myofibroblasts (7), resident fibroblasts have been confirmed as the main source in recent in vivo lineage tracing studies, at least in some vital organs such as the heart (8,9). It is therefore important to identify the regulatory mechanisms in fibroblast cell-type transition. This complex process involves extracellular and cell membrane signaling, cytosolic pathways, epigenetic and transcriptional remodeling, and interactions among these networks (8). The best known fibrogenic signaling pathway is transforming growth factor (TGFβ1). In contrast, the PDGF pathways are less well understood (8). In particular, the PDGF-AA homodimer (hereafter denoted as AA), which selectively activates PDGFRα, is inadequately explored relative to PDGF-BB, which activates both PDGFRα and PDGFRβ (10).

In the present study, we investigated a possible PLK regulation of vascular adventitial fibroblast cell-type transition in the setting of AA-stimulated PDGFRα activation. We focused primarily on the divergent PLK member (PLK4) (3) and included PLK1, the representative member of the PLK family (11). We found that PLK4 inhibition constrained the rat aortic fibroblast proliferative/migratory behaviors and the nuclear activity of serum response factor (SRF), a master transcription factor (8). The latter finding was somewhat surprising, given that PLK4 is deemed to be centriole specific and cytosol localized. We also revealed that PDGFR and downstream kinase P38 positively regulated PLK4 phosphorylation. In pursuit of the transcriptional regulators of PLK4, we identified BRD4 (a bromodomain/extraterminal family member) as an epigenetic determinant of PLK4 expression. Thus, our results reveal a noncanonical function of PLK4. Furthermore, we observed an effect of PLK4 inhibition on attenuating vascular fibrosis in a rat artery injury model.

## Methods

### Animals and ethics statement

Male Sprague-Dawley rats were purchased from Charles River Laboratories (Wilmington, Massachusetts), housed and fed under standard conditions, and used for in vivo experiments at body weights of 300–330 g. All animal studies conformed to the Guide for the Care and Use of Laboratory Animals (National Institutes of Health), and protocols were approved by the Institutional Animal Care and Use Committee. Isoflurane general anesthesia was applied during surgery (through inhaling, at a flow rate of 2 l/min), and buprenorphine was subcutaneously injected (0.03 mg/kg, ∼0.01 mg/rat) after the surgery. Animals were euthanized in a chamber gradually filled with CO_2_.

### Rat aortic adventitial fibroblast cell culture, induction of cell-type transition, and pretreatment with inhibitors

Primary aortic adventitial fibroblasts were isolated from 6–8-week-old male Sprague-Dawley rats. For cell expansion, the culture was maintained at 37°C/5% CO_2_ in Complete Fibroblast Medium (cat. M2267; Cell Biologics, Chicago, Illinois) containing growth factor supplement and 10% fetal bovine serum (FBS; cat. 6912, Cell Biologics); 0.25% Trypsin-EDTA solution (cat. 25200114; Life Technologies, Carlsbad, California) was used for cell detachment. The fibroblast cells at or before passage 5 were used for experiments (Supplemental Figure S1). For induction of fibroblast cell-type transition, cells were first starved overnight in Fibroblast Basal Medium (cat. 2267b, Cell Biologics) that contained no FBS, and then stimulated with 60 ng/mL AA (rat recombinant; R&D Systems, Minneapolis, Minnesota). Time lengths of stimulation are specifically indicated in each figure. In the experiments using various inhibitors (Supplemental Table S1), before adding AA cells were pretreated with an inhibitor for 2 h (at an indicated concentration) or vehicle control (equal volume of dimethylsulfoxide [DMSO]; Sigma-Aldrich, St. Louis, Missouri).

### Cell proliferation and migration assays

Cell viability assay was performed using the CellTiter-Glo kit (cat. G7571; Promega, Madison, WI), as we previously reported (12). Briefly, 72 h after AA treatment, plates were decanted and refilled with 50 μl CellTiter-Glo reagent and 50 μl phosphate-buffered saline solution (PBS) per well. Plates were incubated at room temperature for 20 minutes and then read in a FlexStation 3 Benchtop Multi-Mode Microplate Reader (Molecular Devices, Sunnyvale, California) with the use of a 250-ms integration.

Cell migration was measured using the scratch assay following our previous report (12). Briefly, cells were cultured to 90% confluence in 6-well plates and then starved for 24 h in Fibroblast Basal Medium. A sterile pipette tip was used to generate a ∼1 mm cell-free gap. Dislodged cells were washed away with PBS. Plates were then refilled with Fibroblast Basal Medium containing 20 ng/ml AA and no FBS and incubated for 24 h. For pretreatment before AA stimulation, an inhibitor or vehicle control (equal amount of DMSO) was incubated with the cells for 2 hours or as otherwise specified. For illumination of the cells, Calcein-AM was added (final 2 μmol/l) and incubated for 10 min at the end of the AA treatment, and images were taken after 3 washes with PBS. Cell migration was quantified with the use of Image J (National Institutes of Health, Bethesda, MD) based on the width of the cell-free gap.

### Western blotting to assess protein levels

Western blot analysis was done as we previously described (12). Briefly, cells were harvested and lysed on ice in the RIPA buffer (cat. 89900; Thermo Fisher) that includes a protease and phosphotase inhibitor cocktail (cat. 78441; Thermo Fisher). Cell lysates were quantified for protein concentrations with the use of the Bio-Rad DC Protein Assay kit (cat. 5000112) and loaded on a 10% sodium dodecyl sulfate–polyacrylamide gel electrophoresis gel. The full primary antibody list is presented in Supplemental Table S2. Beta-actin or GAPDH was used for loading control. The signals from primary antibodies were amplified by means of horseradish peroxidase (HRP)–conjugated immunoglobulin G (Bio-Rad) and illuminated with the use of Pierce ECL Western Blotting Substrates (Thermo Fisher). Blot images were immediately recorded with Azure C600 Imager (Azure Biosystems). Protein band densitometry was quantified with the use of ImageJ and normalized to loading control for statistical analysis.

### Gene silencing with siRNAs

Small interfering (si) RNAs were ordered from Thermo Fisher (sequences listed in Supplemental Table S3). Cells were grown to ∼70% confluence in 6-well plates in Complete Fibroblast Medium. A gene-specific siRNA was added to transfect fibroblast cells overnight with the use of the RNAi Max reagent (cat. 13778150; Thermo Fisher). Cells then recovered in the complete medium for 24 hours. For induction of fibroblast cell-type transition, the culture was changed to Fibroblast Basal Medium and incubated overnight before AA stimulation.

### Quantitative real-time polymerase chain reaction to determine mRNA levels

We followed the method described in our previous report (12). Briefly, total RNA was isolated from cultured cells with the use of the Trizol reagent (Invitrogen, Carlsbad, California). Potential contaminating genomic DNA was removed by using gDNA Eliminator columns provided in the kit. RNA was quantified with a Nanodrop NP-1000 spectrometer, and 1 μg was used for the first-strand cDNA synthesis. Quantitative real-time polymerase chain reaction was then performed with Quant Studio 3 (Applied Biosystems, Carlsbad, California). The house keeping gene GAPDH was used for normalization. Each cDNA template was amplified in triplicate PerfeCTa SYBR Green SuperMix (Quantabio, Beverly, Massachusetts) with the gene-specific primers listed in Supplemental Table S4.

### Luciferase reporter assay for SRF transcriptional activity

We followed the manufacturer’s instruction and our recently reported protocol (13). Briefly, the pGL4.34 vector plasmid containing the CArG box (SRF response element) was purchased from Promega (cat. E1350). An empty vector was generated by removing the SRF response element. Cells were transfected with the empty vector (control) or pGL4.34 using jetPRIME Transfection Reagent (cat. 114-07; Polyplus-Transfection, New York, New York). Positively transfected cells (HEK293) were selected with hygromycin B (cat. 10687010; Thermo Fisher), seeded in 24-well plates at a density of 20,000 cells/well, and grown for 6 hours. Cells were treated with vehicle (DMSO) or 1 μmol/l centrinone-B (CenB) for 2 h and then lysed in Bright-Glo (cat. 2610; Promega), and luminescence was read in the FlexStation 3 Benchtop Multi-Mode Microplate Reader.

### Lentiviral constructs for the expression of PLK4 wild type and mutants

The cDNA of full-length wild type (WT) PLK4 gene (coding sequence of NM_011495.2) was reverse transcribed from mRNA, which was extracted from the C3H (mouse embryo fibroblast) cell line with the use of Trizol reagents. The PLK4 gene was cloned into the Lenti-puro vector (#39481; Addgene, Watertown, Massachusetts) in fusion with a 2×FLAG tag at the N-terminus. To generate PLK4 mutants, the PLK4 gene with a mutation was subcloned into the same vector. The primers used in this study are listed in Supplemental Table S5. Lentivirus was produced using HEK293FT cells (Invitrogen) with the second-generation packaging system including pSPAX2 (plasmid #12260, Addgene) and pMD2.G (plasmid #12259, Addgene). Lentivirial titer was determined using Lenti-X GoStix Plus (TakaRa, Mountain View, California), which measures viral RNA content. For the expression of PLK4 WT and mutants, lentivirus was transduced into the C3H cell line and incubated for 2 days in high-glucose Dulbecco Modified Eagle Medium containing 10% FBS, and 1 μg/mL puromycin was included for selection.

### Model of rat carotid artery injury and periadventitial administration of PLK4 inhibitor

To induce adventitial fibrosis, balloon angioplasty injury was performed in rat common carotid arteries as we previously described (12). Briefly, rats were anesthetized with isoflurane (5% for inducing and 2.5% for maintaining anesthesia). A longitudinal incision was made in the neck to expose carotid arteries. A 2-F balloon catheter (Edwards Lifesciences, Irvine, California) was inserted through an arteriotomy on the left external carotid artery and advanced into the common carotid artery. To produce arterial injury, the balloon was inflated at a pressure of 2 atm and withdrawn to the carotid bifurcation and this action was repeated 3 times. The external carotid artery was then permanently ligated and blood flow was resumed. Immediately after balloon injury, a PLK4 inhibitor (centrinone-B, 100 μg/rat) or DMSO control dissolved in a mix of 2 thermosensitive hydrogels was administered around the adventitia of injured arteries. The hydrogel mix (total 400 μL) contained equal volume of 20% AK12 (PolySciTech; AKINA, West Lafayette, Indiana) and 25% Pluronic gel (Sigma-Aldrich). One week after balloon injury, common carotid arteries were collected from anesthetized animals after perfusion fixation at a physiological pressure of 100 mm Hg. Throughout the surgery, the animal was kept anesthetized via isoflurane inhaling at a flow rate of 2 l/min. Buprenorphine was subcutaneously injected (0.03 mg/kg, ∼0.01 mg/rat) after the surgery. Animals were euthanized in a chamber gradually filled with CO_2_.

### Morphometric analysis

Cross-sections (5 μm thick) were excised from rat common carotid arteries embedded in paraffin blocks. Sections were stained for collagen and morphometric analyses with the use of a Masson's trichrome approach (14) with reagents from Abcam (cat. ab150686). Collagen was stained blue, and smooth muscle actin (media layer) was stained red. Fibrosis was assessed by 2 parameters in parallel: the thickness and collagen content of the adventitia layer, which was distinguishable from other tissue layers by distinct colors. Collagen content was measured as blue stain intensity normalized to the artery overall size (length of external elastic lamina). Planimetric parameters for assessing intimal hyperplasia (intima/media area ratio) were measured following our previous report (12): area inside external elastic lamina (EEL area), area inside internal elastic lamina (IEL area), lumen area, intima area (IEL area − lumen area), and media area (EEL area − IEL area). All measurements were performed in ImageJ by a student blinded to treatment groups. The data from all 3–5 sections were pooled to generate the mean for each animal. The means from all the animals in each treatment group were then averaged, and the standard error of the mean was calculated.

### Immunostaining on artery tissue sections

Fluorescent immunostaining was performed following our published protocol (15). Briefly, artery sections were incubated with the antibody for vimentin (sc-373717, 1:100; Santa Cruz, Dallas, Texas) for 12 h and rinsed at least 3 times. The sections were then incubated with an antimouse secondary antibody conjugated with Alexa Fluor 594 (A-21203; Invitrogen) and rinsed. Vimentin was then visualized with the use of fluorescence microscopy (EVOS M7000; Thermo Fisher Scientific). Antibodies are presented in Supplemental Table S6. For quantification, at least 3 immunostained sections from each animal were used. Fluorescence intensity in the adventitia layer of each image field was quantified with the use of ImageJ software and normalized to the number of 6-diamino-2-phenylindole–stained nuclei. The data values from all sections were pooled to generate the mean for each animal. The means from all animals in each group were then averaged, and the final mean ± SEM was calculated.

### Statistical analysis

Differences in measured variables between experimental conditions were assessed by 1-way analysis of variance ANOVA with post hoc Bonferroni test or Student *t*-test (comparison between only 2 conditions), as specifically stated in each figure legend. A p value < 0.05 was considered to be statistically significant. Data are presented as the mean ± SEM from at least 3 independent repeated experiments. Statistics and graphical data plots were generated with the use of GraphPad Prism v. 5.0 for Windows.

## Results

### PLK4 inhibition blocks cell-type transition of rat aortic adventitial fibroblasts

**Figure 1:**
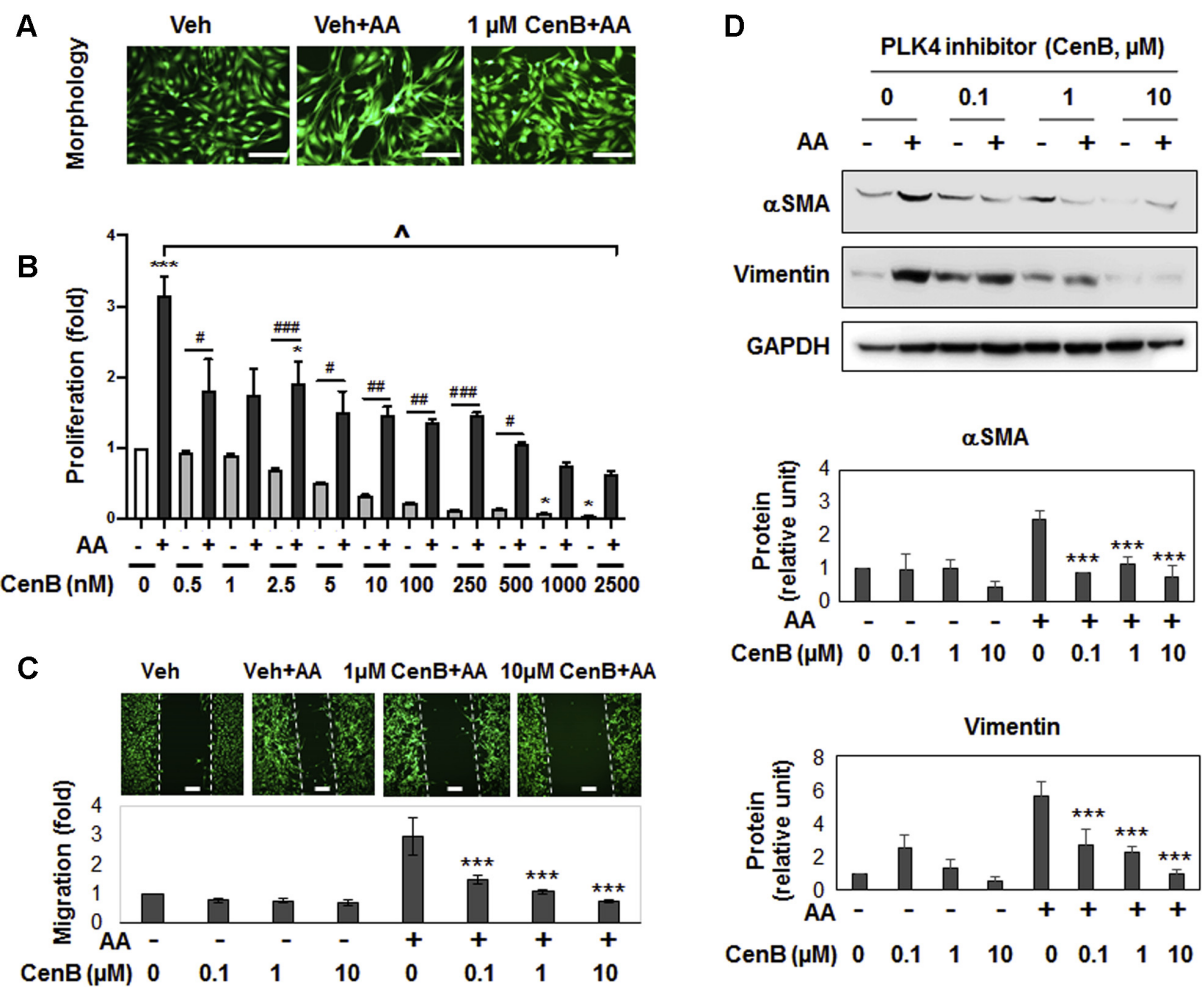
PLK4 Inhibition Blocks Cell-Type Transition and αSMA Expression of Rat Adventitial Fibroblasts Rat primary adventitial fibroblasts were cultured in the complete medium, starved in the basal medium overnight (see Methods), and pretreated for 30 min with vehicle (equal amount of DMSO) or the PLK4-selective inhibitor CenB at indicated concentrations, followed by stimulation with 60 ng/ml AA. Cells were harvested 24 h after stimulation (or as specifically indicated) for various assays. **(A)** Morphology. Cells were (or were not) stimulated by AA for 24 h without or with pretreatment (1 μmol/l CenB). Green fluorescent calcein was used to illuminate cell morphology. **(B)** Proliferation. CellTiter-Glo assay was performed after 72 h stimulation (by AA or solvent) without or with pretreatment with CenB at increasing concentrations. **(C)** Migration (scratch assay). Cells were (or not) stimulated by AA for 24 h without or with pretreatment (1 or 10 μmol/l CenB). Calcein was used to illuminate the cells. **(D)** Western blots of αSMA and vimentin. Protein band densitometry was normalized to loading control (β-actin) and then to the basal condition (DMSO, no AA), and finally quantified as fold change. Fold changes from at least 3 independent experiments were averaged, and mean ± SEM was calculated. One-way ANOVA/Bonferroni test: #p < 0.05; ##p < 0.01; ###p < 0.001. ∗p < 0.05; ∗∗∗p < 0.001 compared with vehicle control without AA **(B)** or with AA **(C, D)**; ^ˆ^p < 0.05 between vehicle + AA and all other conditions in **(B)**. αSMA = α-smooth muscle actin; AA = platelet-derived growth factor; ANOVA = analysis of variance; CenB = centrinone-B; DMSO = dimethylsulfoxide; PLK = Polo-like kinase.

We used PDGF-AA, which preferentially activates PDGFRα versus PDGFRβ (10) to stimulate fibroblast cell-type transition. As shown in Figures 1A to 1C), treating cells with AA induced an elongated morphology and ∼3-fold increases of proliferation and migration. We then investigated the effect of manipulating PLK4 on this AA-induced process. We took advantage of recent progress in developing highly selective PLK inhibitors that provide powerful tools for deciphering PLK4 functions. Centrinone-B (CenB) is a novel PLK4-selective inhibitor (Ki = 0.6 nmol/l) with very low affinities for other PLK and non-PLK kinases (1). Pretreatment of rat aortic fibroblasts with CenB abrogated AA-stimulated proliferation and migration in a concentration-dependent fashion (Figures 1B and 1C), and 100 nmol/L CenB showed a significant functional effect. Furthermore. AA-stimulated up-regulation of αSMA and vimentin proteins (2.5–5-fold) was abolished by pretreatment with CenB (Figure 1D). PLK4 inhibition with CenB also effectively attenuated TGFβ1-stimulated vimentin upregulation (Supplemental Figure S2). Because this study was focused on PDGFRα pathways, we used AA as a stimulant throughout.

**Figure 2:**
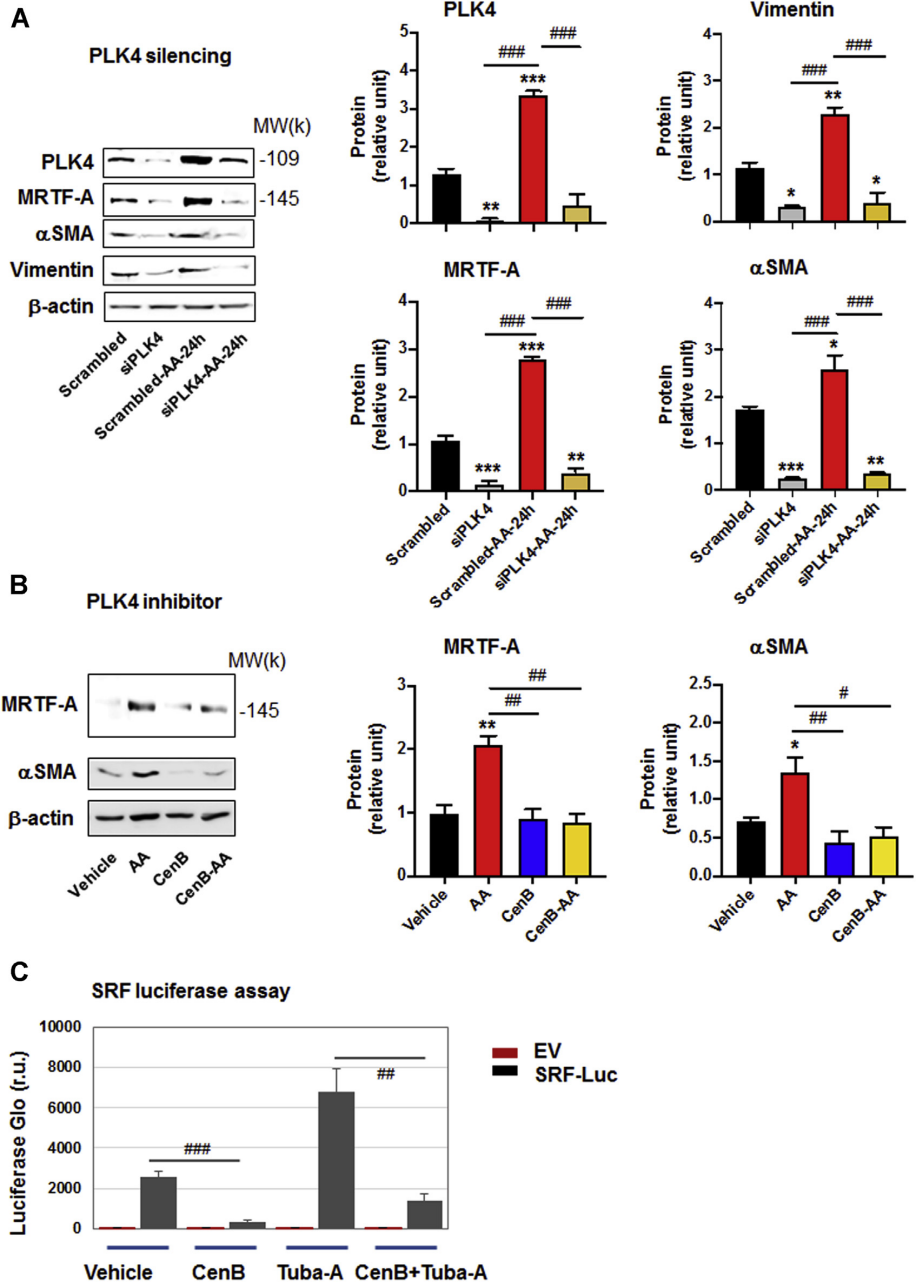
PLK4 Inhibition Reduces MRTF-A Protein and SRF Activity **(A)** Effect of PLK4 silencing on MRTF-A. Cells were transfected with scrambled or PLK4-specific siRNA for 48 h in the complete medium, starved overnight, and then stimulated with solvent or AA for 24 h before harvest for Western blotting. **(B)** Effect of PLK4 inhibition on MRTF-A. Cells were pretreated with 1 μmol/l CenB (or vehicle) and then stimulated with solvent or AA for 48 h before harvest for Western blotting. Mean ± SEM, n = 3 experiments, 1-way ANOVA/Bonferroni test: #p < 0.05; ##p < 0.01; ###p < 0.001. ∗p < 0.05. ∗∗p < 0.01; ∗∗∗p < 0.001 compared with the control of scrambled siRNA or vehicle without AA stimulation (**the first bar in each plot**). **(C)** Luciferase reporter assay of SRF transcriptional activity. Cells were transfected with the empty vector control or the SRF-luciferase vector, followed by luminescence reading. The condition with 5 μmol/l tubastatin-A, a histone deacetylase 6 inhibitor and a novel SRF stimulator (13), served as a positive control. Mean ± SEM, n ≥3 experiments, Student *t*-test: ##p < 0.01; ###p < 0.001 (between 2 **gray bars**). MRTF-A = myocardin-related transcription factor A; SRF = serum response factor; other abbreviations as in Figure 1.

Importantly, we confirmed the PLK4 functional specificity. We did this by silencing PLK4 and showing that αSMA and vimentin protein levels were reduced (Figure 2A). While it is intuitive that PLK4 as a centriole factor promoted cell proliferation (1), a role for PLK4 in elevating αSMA expression was somewhat unexpected, given PLK4’s canonical association with centrioles in the cytoplasm.

We then investigated the mechanism underlying PLK4-stimulated αSMA expression with αSMA transcription is known to be driven by SRF, a master transcription factor. SRF is itself activated by myocardin-related transcription factor A (MRTF-A), a powerful transcription regulator that shuttles between the cytoplasm and nucleus (13). We therefore investigated the influence of PLK4 on MRTF-A protein and SRF transcriptional activity. We found that while treatment with AA elevated total MRTF-A protein levels, either PLK4 silencing (Figure 2A) or inhibition with CenB (Figure 2B) prevented this elevation. In the presence of CenB, αSMA was substantially reduced in both cytosol and nucleus, and AA could not increase nuclear MRTF-A protein (Supplemental Figure S3). Furthermore, the SRF transcriptional (luciferase) activity was diminished by CenB (Figure 2C). These results revealed that, in rat aortic adventitial fibroblasts, PLK4 plays an important role in SRF activation and αSMA production, at least in part by elevating MRTF-A protein levels.

Taken together, our results indicate that PLK4 regulates fibroblast cell-type transition and, intriguingly, SRF nuclear activity, a function that apparently departs from centriole duplication. To the best of our knowledge, this noncanonical PLK4 function in SRF activation has not been previously reported. It is also noteworthy that pretreatment with CenB largely preserved normal fibroblastic phenotypes (Figure 1) and did not cause obvious cell death even at high (e.g., 10 μmol/l) concentrations, suggesting a low cytotoxicity of this drug.

**Figure 3:**
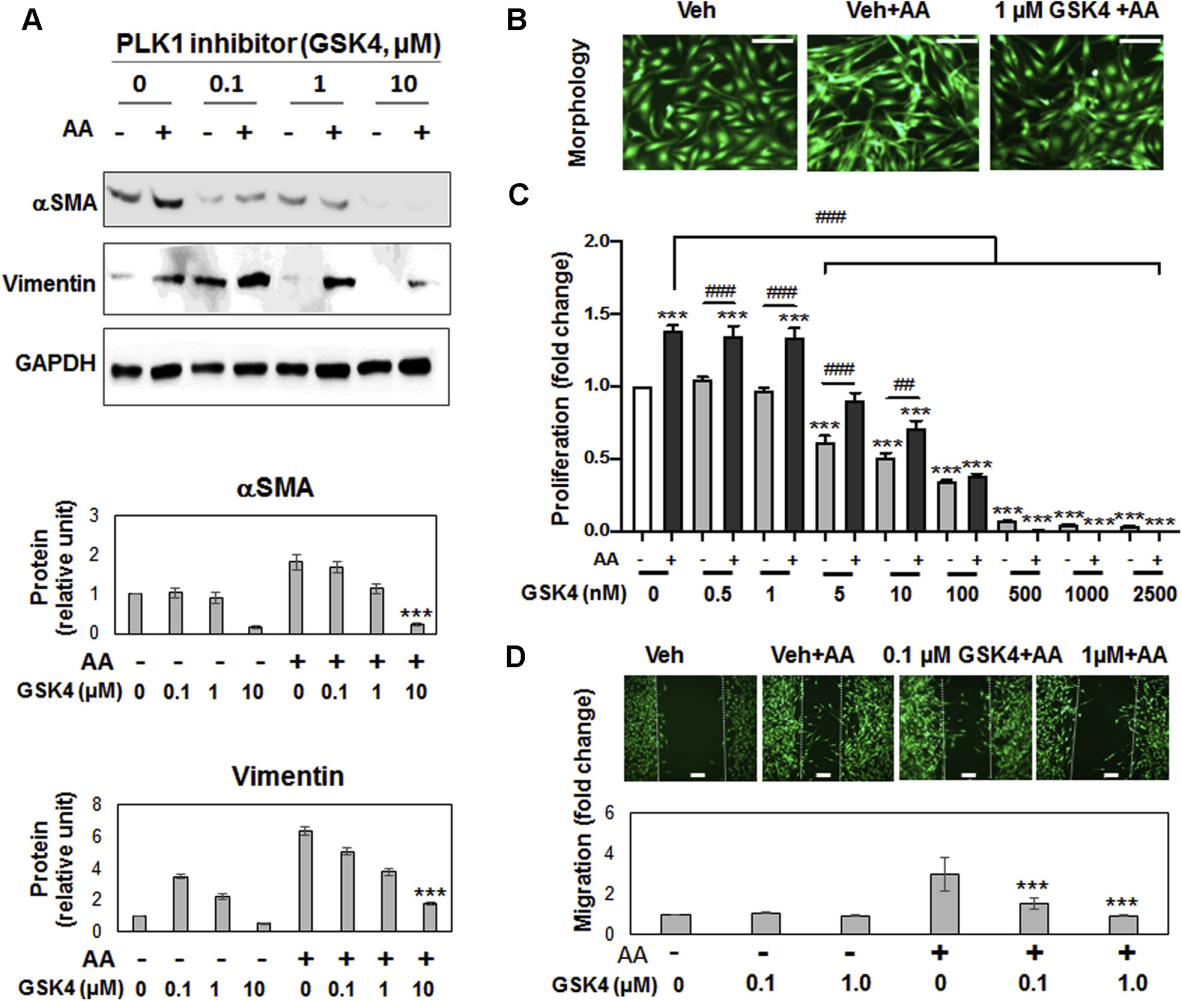
PLK1 Inhibition Blocks PDGF-AA–Stimulated Fibroblast Cell-Type Transition Experiments were performed as described in Figure 1 except that the PLK1-selective inhibitor GSK461364 (GSK4) was used for pretreatment before AA stimulation. **(A)** Western blots of αSMA and vimentin. **(B)** Morphologic comparison. Cells were (or were not) stimulated by AA for 24 h without or with pretreatment (1 μmol/l GSK4). **(C)** Proliferation. CellTiter-Glo assay was performed after 72 h stimulation (by AA or solvent) without or with pretreatment with GSK4 at increasing concentrations. **(D)** Migration (scratch assay). Cells were (or not) stimulated by AA for 24 h without or with pretreatment (0.1 or 1 μmol/l GSK4). Mean ± SEM, n ≥3 experiments, 1-way ANOVA/Bonferroni test: #p < 0.05; ##p < 0.01; ###p < 0.001. ∗p < 0.05; ∗∗∗p < 0.001 compared with vehicle control without AA **(C)** or with AA **(A, D)**. Abbreviations as in Figure 1.

We also determined the effect of PLK1 inhibition on fibroblast phenotypes using the PLK1-selective inhibitor GSK461364 (Ki = 2.2 nmol/l) (16). The result (Figure 3) was similar to that of PLK4 inhibition (Figure 1). However, the PLK1 inhibitor at high concentrations (e.g., 0.5 μmol/l) reduced cell viability to much below the nonstimulated basal level (Figure 3C), consistent with its cytotoxicity (16).

### Blocking PDGFR kinase activity abrogates AA-stimulated PLK4 phosphorylation

Given the profound effect of blocking PLK4 kinase activity on fibroblast cell-type transition, it is important to understand how the activation (phosphorylation) and expression of PLK4 are regulated. Currently there is very limited information available on this topic. In our experimental setting, PDGF-AA activates PDGFRα, a well known “gateway” receptor on the cell surface that, in turn, activates a myriad of intracellular pathways (8,10). However, whether PDGFRα regulates PLK4 was not previously known. We addressed this question with the use of a kinase inhibitor (crenolanib) selective to PDGFRα and β (Ki <10 nmol/l) (17).

**Figure 4:**
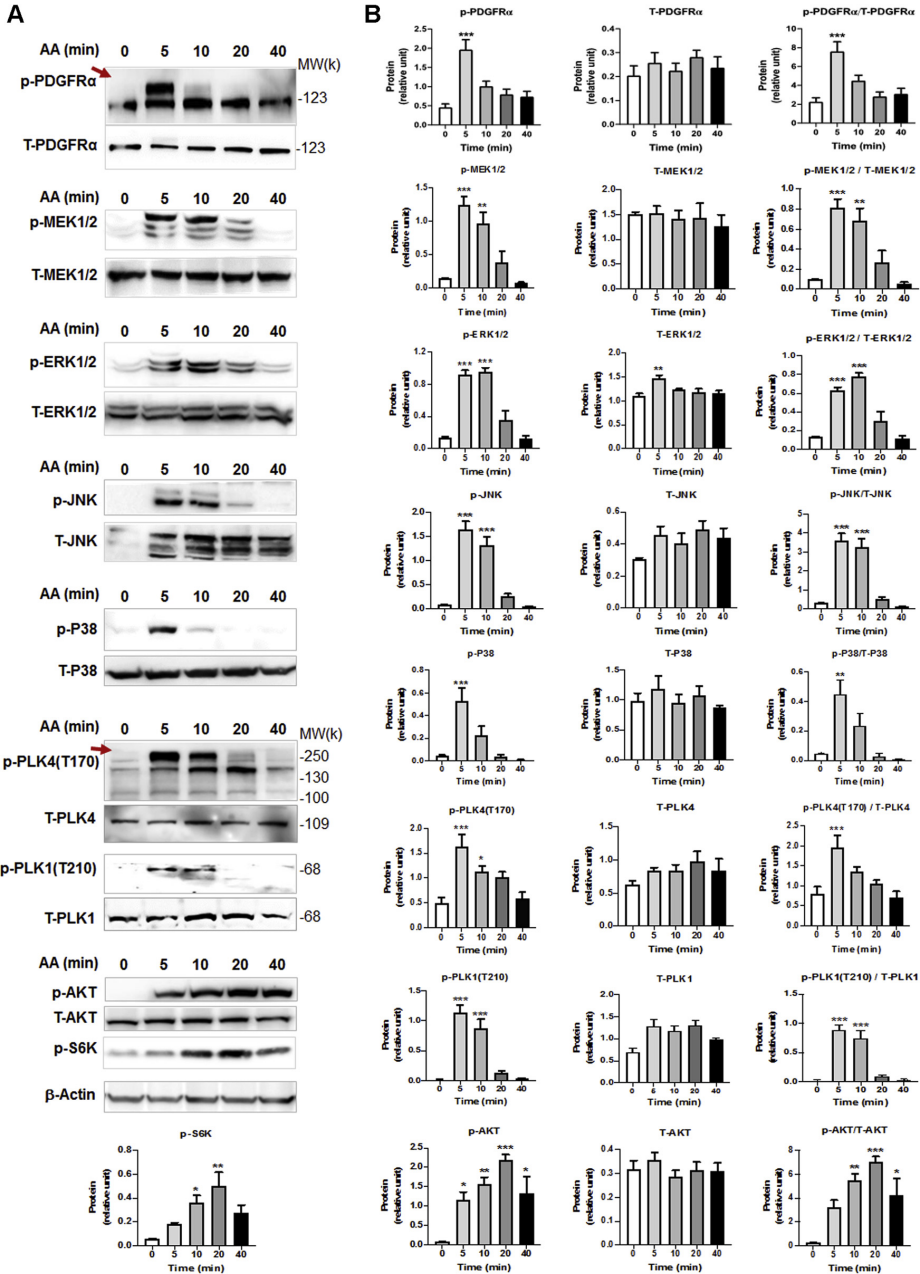
AA Stimulates Rapid PDGFRα and PLK4 Phosphorylation Rat primary adventitial fibroblasts were cultured, starved, and pretreated with vehicle (DMSO) or an inhibitor, as described in Figure 1. The **arrow** points to phospho-protein bands. **(A)** Representative Western blots showing the time course of AA-induced phosphorylation of kinases. Western blots detect a phospho-protein (p-) and its respective total protein (T-). **(B)** Quantification: mean ± SEM, n ≥3 experiments. One-way ANOVA/Bonferroni test: ∗p < 0.05; ∗∗p < 0.01; ∗∗∗p < 0.001 compared with the 0-min control; p-S6K was included as a downstream effector of the AKT pathway. PDGFR = platelet-derived growth factor receptor; other abbreviations as in Figure 1.

**Figure 5:**
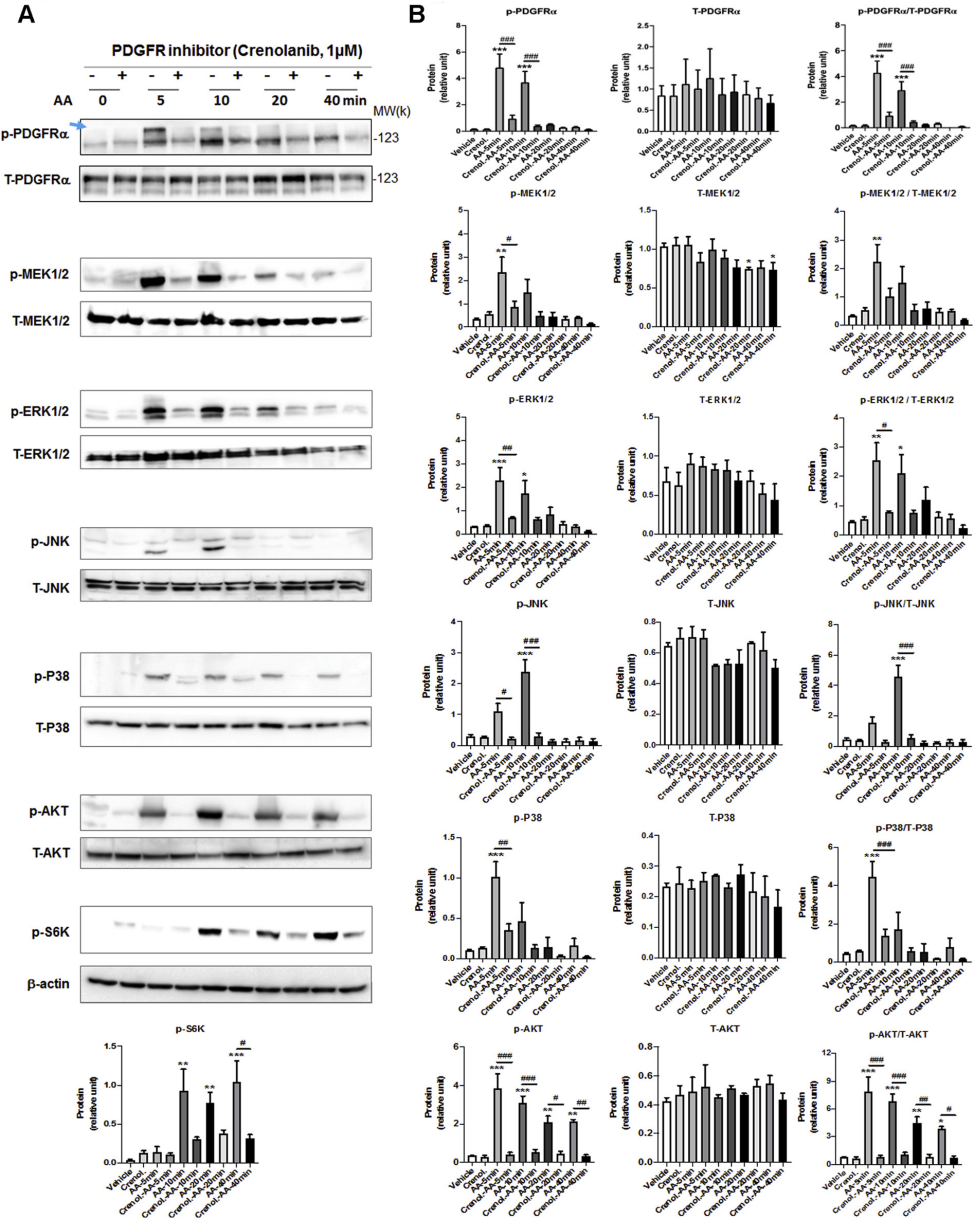
PDGFR Inhibition Blocks the Phosphorylation of Downstream Kinases Rat primary adventitial fibroblasts were cultured, starved, and pretreated with vehicle (DMSO) or PDGFR-selective inhibitor crenolanib (1 μmol/l, 30 min). The **arrow** points to the bands of phospho-PDGFRα that were sensitive to both AA stimulation and inhibitor pretreatment. **(A)** Representative Western blots showing blockade of AA-induced kinase activation by the PDGFR-selective inhibitor crenolanib. p- = phospho-protein; T- = total protein. **(B)** Quantification: mean ± SEM, n ≥3 experiments. One-way ANOVA/Bonferroni test: #p < 0.05; ##p < 0.01; ###p < 0.001. ∗p < 0.05; ∗∗p < 0.01; ∗∗∗p < 0.001 compared with the vehicle control without AA stimulation **(the first bar in each plot)**. Abbreviations as in Figures 1 and 4.

**Figure 6:**
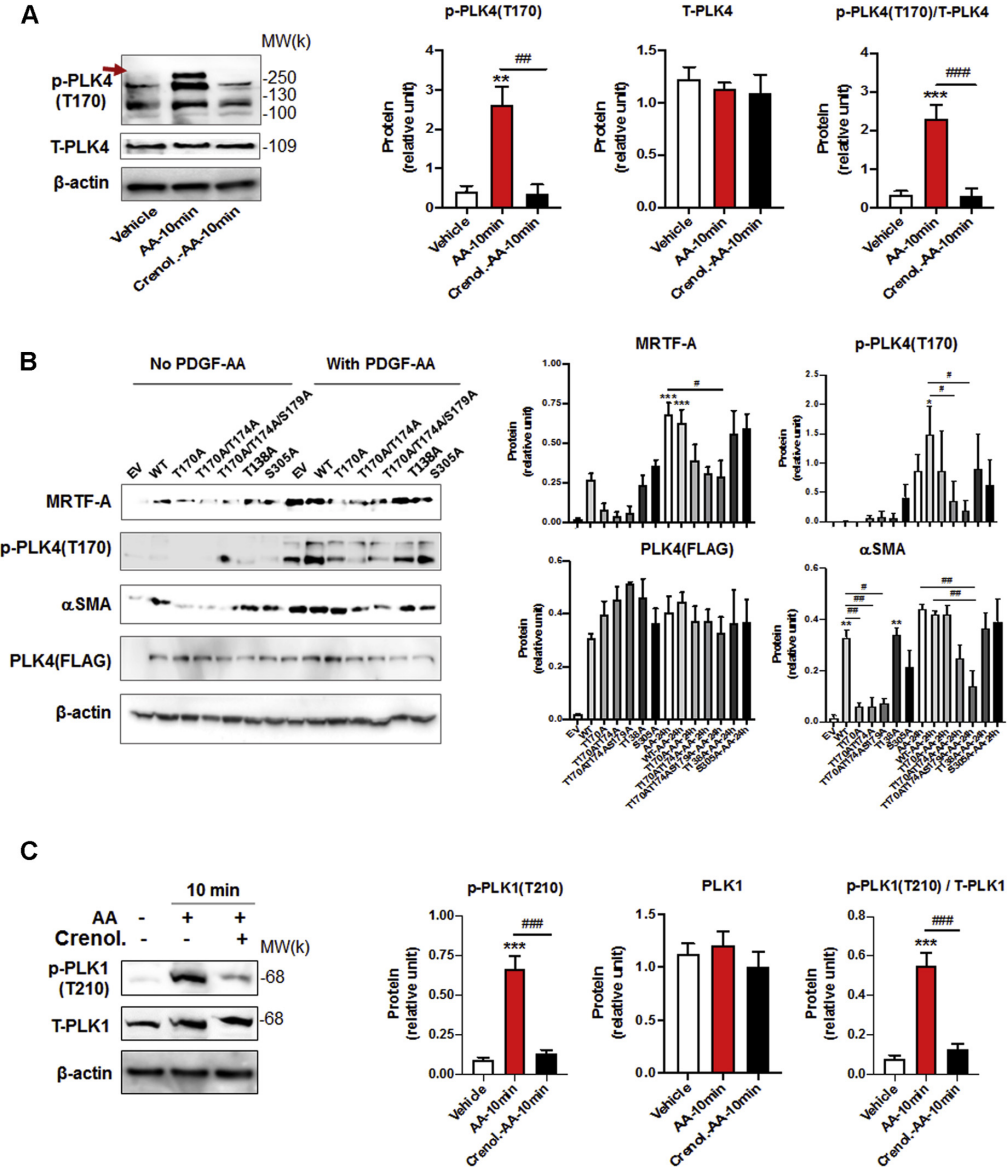
PDGFR Inhibition Blocks PLK4 Phosphorylation **(A)** Inhibition of PLK4 phosphorylation by the PDGFR-selective inhibitor crenolanib. Rat primary adventitial fibroblasts were cultured, starved, and pretreated with vehicle (DMSO) or crenolanib (1 μmol/l, 30 min), and then stimulated with AA (60 ng/ml) for 10 min before harvest for Western blotting. The **arrow** points to the band of phospho-PLK4. **(B)** PLK4 mutation at T170 inhibits PLK4 function. Fibroblast cells (C3H cell line) transduced with lentivirus to express the empty vector control (EV) or PLK4 wild type (WT) or mutants were selected with puromycin, starved overnight and then stimulated with solvent or AA (60 ng/ml). Exogenous PLK4 expression levels were monitored by Western blotting against the FLAG tag. **(C)** Inhibition of PLK1 phosphorylation by the PDGFR-selective inhibitor crenolanib. Rat primary adventitial fibroblasts were cultured, starved, and pretreated with vehicle (DMSO) or crenolanib (1 μmol/l, 30 min), and then stimulated for 10 min before harvest for Western blotting. Quantification: mean ± SEM, n ≥3 experiments, 1-way ANOVA/Bonferroni test: #p < 0.05; ##p < 0.01; ###p < 0.001. ∗p < 0.05; ∗∗p < 0.01; ∗∗∗p < 0.001 compared with the basal control **(the first bar in each plot)**. Abbreviations as in Figures 1 and 4.

AA treatment of rat fibroblasts led to the rapid and brief increase in phosphorylation of PDGFRα (at Y754, commonly seen as an upper band) (17). Specifically, we observed increased phosphorylation within 5 min that declined to the basal level in 20 min (Figure 4). AA also stimulated phosphorylation of MEK, ERK, JNK, and P38 with time course similar to that for PDGFRα, consistent with published reports from other cell types (18,19). Interestingly, treatment with AA activated PLK4 (phosphorylation at T170) (20) and PLK1 (phosphorylation at T210) (21) in the same time scale as for PDGFRα (Figure 4). Importantly, while the PDGFR-selective inhibitor crenolanib blocked AA-induced activation of PDGFRα as well as MEK/ERK, JNK, P38, and AKT (and its downstream effector S6K), validating the inhibitor functionality (Figure 5), this inhibitor also abrogated AA-stimulated phosphorylation of PLK4 and PLK1 (Figures 6A and 6C), placing them downstream of PDGFRα. Of note, the phospho-PLK4 bands most sensitive to AA and crenolanib ran at a high position on the blot. Inasmuch as activated PLK4 dimerizes tightly to stabilize the protein (22), it is tempting to speculate that a transient oligomer may form before its disassembly and degradation. Indeed, phospho-PLK4–positive bands higher than an expected mobility position have also been reported elsewhere (23,24), and recent crystal structures showed a strand-swapped dimer of dimers of the PLK4 PB3 domain (4), which is unique to PLK4 and absent in other PLKs.

### Abrogating PLK4 phosphorylation via mutagenesis reduces MRTF-A and αSMA production

To further delineate the importance of PLK4 phosphorylation in the fibroblast phenotypic transition, we generated a series of PLK4 mutants, at T170 and its adjacent sites (T174 and S179) and other candidate sites of PLK4 phosphorylation (T138 and S305) (24,25). Approximately even protein expression levels of PLK4 WT and mutants are shown in Figure 6B. With basal-level activation (no AA), elevating PLK4 WT markedly increased MRTF-A protein. The T170A mutant, T170A/T174A double mutant, and T170A/T174A/S179A triple mutant did not show this effect, indicative of disrupted PLK4 function due to mutation at T170. In contrast, the effect of PLK4 gain-of-function on MRTF-A protein levels was not affected by either T138A or S305A. This pattern of result obtained from the mutations at different PLK4 sites was also observed in the presence of AA stimulation (Figure 6B). Using αSMA as readout, the result was similar to that observed with MRTF-A. Importantly, we confirmed that the mutation at T170 (single, double, or triple mutation), but not that at T138 or S305, significantly hampered AA-stimulated PLK4 phosphorylation (Figure 6B). The double and triple mutation appeared to have enhanced impact on PLK4 phosphorylation and MRTF-A/αSMA protein levels, although the differences did not reach statistical significance. Nevertheless, the PLK4 loss-of-function and gain-of-function results (Figures 2 and 6) together indicate an important role for PLK4 phosphorylation (especially at T170) in mediating AA-stimulated fibroblast phenotypic transition.

### PLK4 phosphorylation is regulated by PDGFR downstream kinase activity but not vice versa

**Figure 7:**
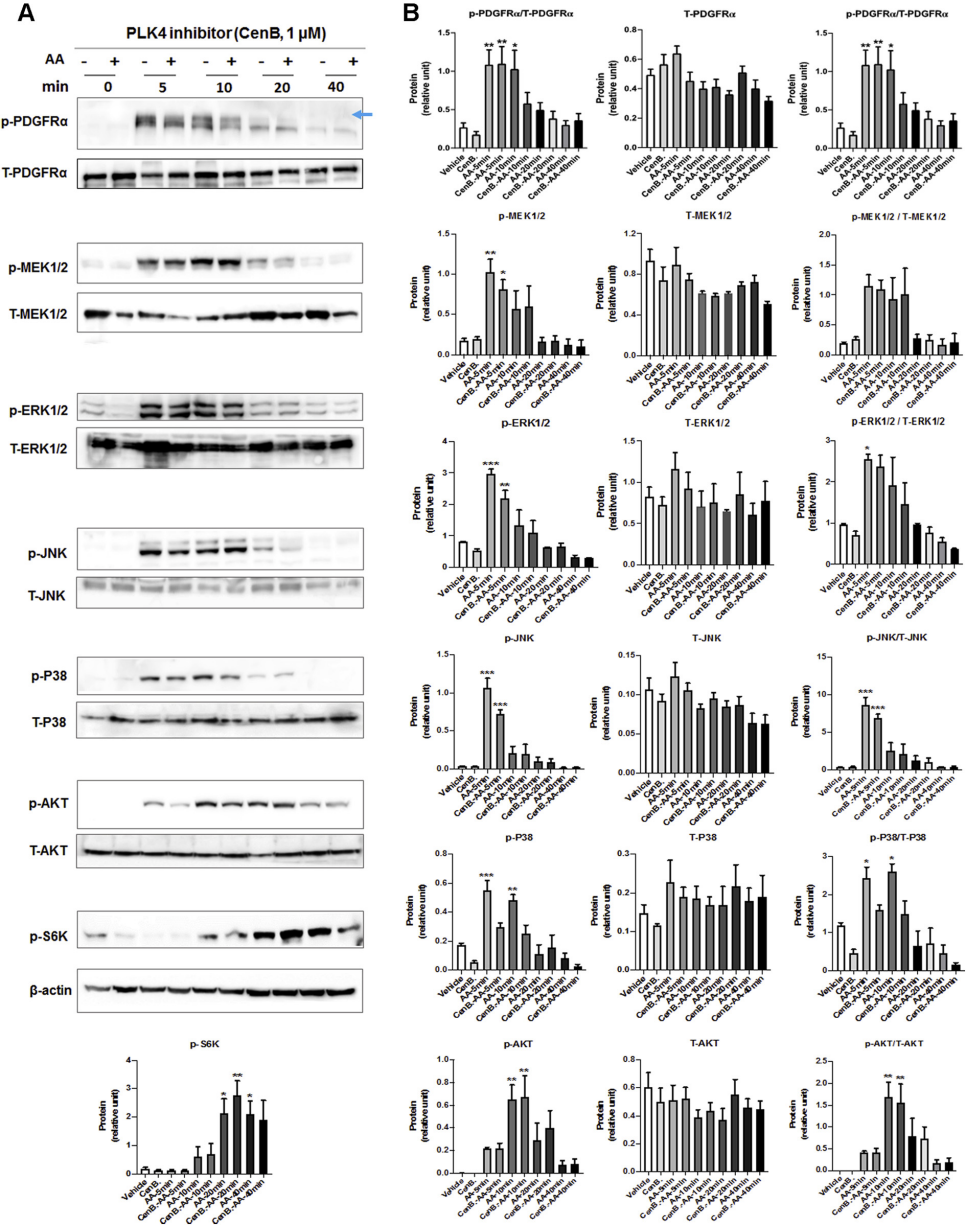
Effect of PLK4 Inhibition on Phosphorylation of PDGFR Downstream Kinases Rat primary adventitial fibroblasts were cultured, starved, and pretreated with vehicle (DMSO) or PLK4-selective inhibitor CenB (1 μmol/l, 30 min), and then stimulated with solvent or AA (60 ng/ml) before harvest for Western blotting. The **arrow** points to the bands of phospho-PDGFRα that were sensitive to AA stimulation. **(A)** Representative Western blots showing the time course of AA-induced phosphorylation of kinases. p- = phospho-protein; T- = total protein. **(B)** Quantification: mean ± SEM, n ≥3 experiments, 1-way ANOVA/Bonferroni test: ∗p < 0.05; ∗∗p < 0.01; ∗∗∗p < 0.001 compared with the basal control (vehicle, no AA stimulation, the first bar in each plot). Abbreviations as in Figures 1 and 4.

**Figure 8:**
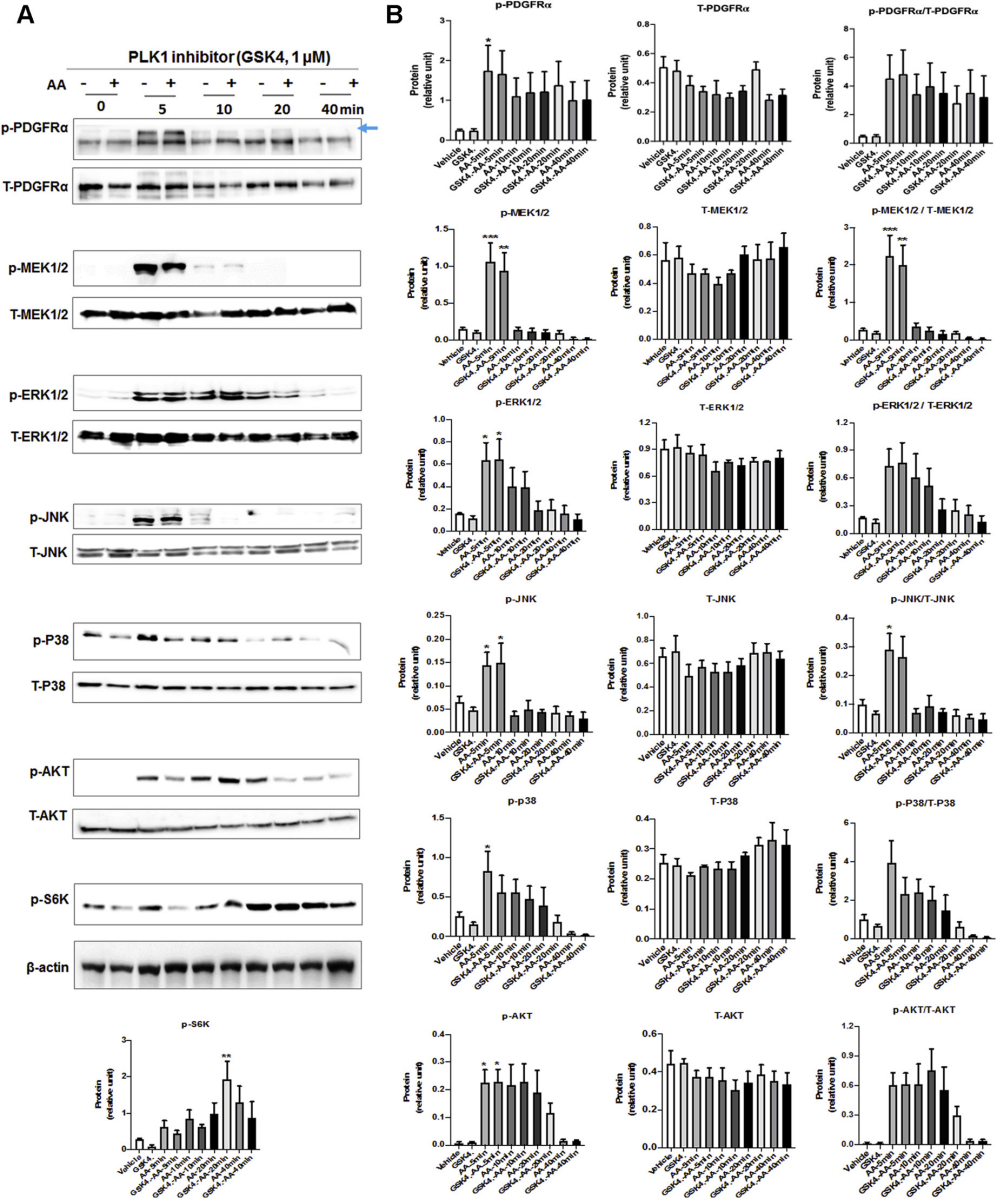
Effect of PLK1 Inhibition on Phosphorylation of PDGFR Downstream Kinases Rat primary adventitial fibroblasts were cultured, starved, and pretreated with vehicle (DMSO) or PLK1-selective inhibitor GSK461364 (GSK4; 1 μmol/l, 30 min) and then stimulated with solvent or AA (60 ng/ml) before harvest for Western blotting. The **arrow** points to the bands of phospho-PDGFRα that were sensitive to AA stimulation. **(A)** Representative Western blots showing the time course of AA-induced phosphorylation of kinases. p- = phospho-protein; T- = total protein. **(B)** Quantification: mean ± SEM, n ≥3 experiments, 1-way ANOVA/Bonferroni test: ∗p < 0.05; ∗∗p < 0.01; ∗∗∗p < 0.001 compared with the basal control (vehicle, no AA stimulation, the first bar in each plot). Abbreviations as in Figures 1 and 4.

**Figure 9:**
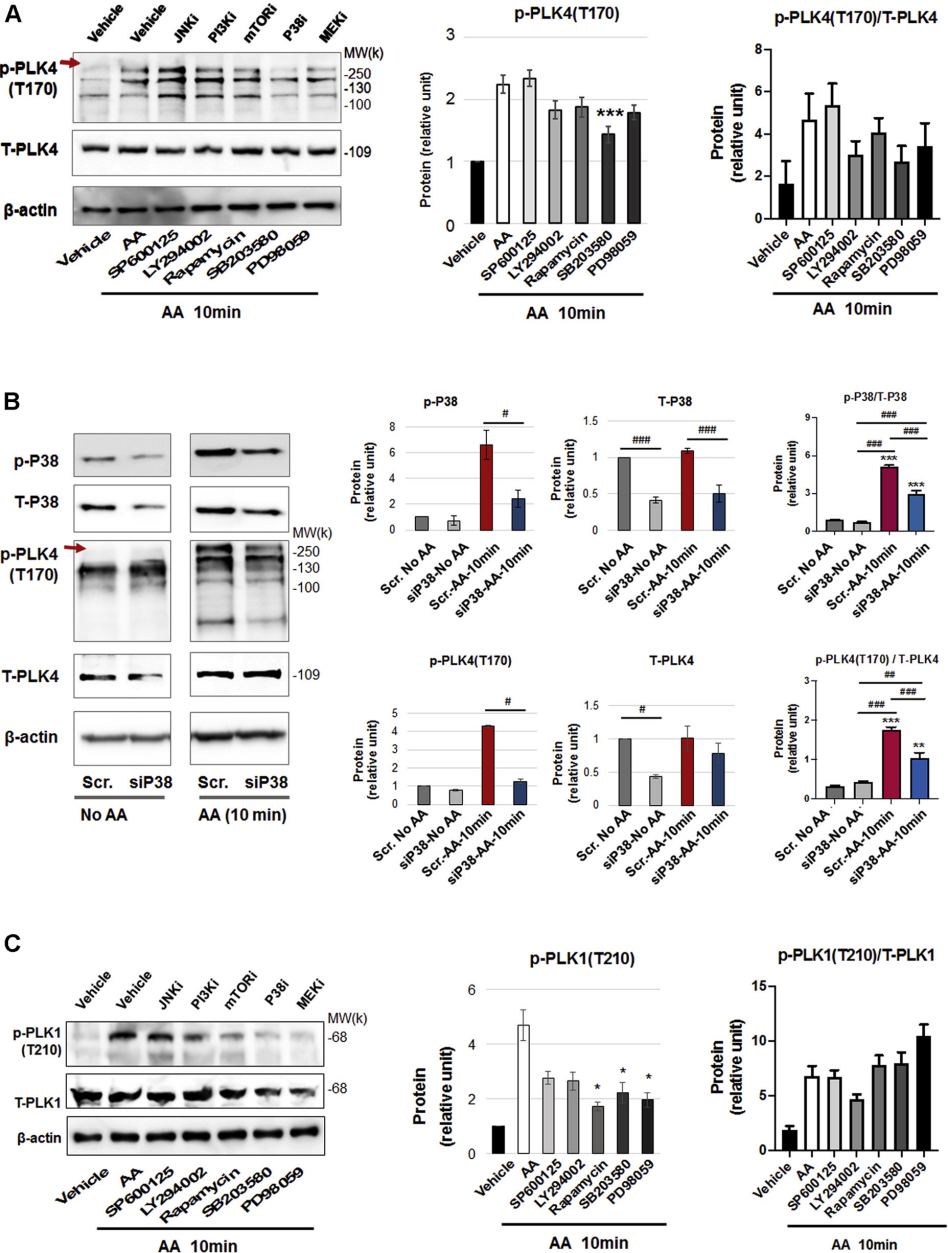
Effect of Inhibition of PDGFR Downstream Kinases on PLK4 Phosphorylation **(A)** P38 inhibition mitigates PLK4 phosphorylation. Rat primary adventitial fibroblasts were cultured, starved, and pretreated with vehicle (DMSO) or a kinase inhibitor (5 μmol/l, 30 min), and then stimulated with AA (60 ng/ml) for 10 min before harvest for Western blotting. The **arrow** points to the bands of phospho-PLK4. Inhibitors: JNK (SP600125), PI3K (LY294002), mTOR (rapamycin). P38 (SB230580), and MEK/ERK (PD98059). **(B)** P38 silencing mitigates PLK4 phosphorylation. Rat primary adventitial fibroblasts were transfected with scrambled (Scr) or P38-specific siRNA (siP38), cultured, starved, and then stimulated with AA (60 ng/ml) for 10 min before harvest for Western blotting. **(C)** Effect of P38 inhibition on PLK1 phosphorylation. Experiments were performed as described in **(A)** except for detection of p-PLK1 (T210). Quantification: mean ± SEM, n ≥3 experiments, 1-way ANOVA/Bonferroni test: #p < 0.05; ##p < 0.01; ###p < 0.001. ∗p < 0.05; ∗∗p < 0.01; ∗∗∗p < 0.001 compared with the vehicle control (with AA, the **white bar** in **A** and **C**) or the scrambled siRNA control without AA (the **first bar** of plot in **B**). Abbreviations as in Figures 1 and 4.

To delineate the position of PLK4 in the signaling cascade downstream of PDGFR, we first tested whether PLK4 blockade affects the activation of the MAPK and AKT pathways, because they were previously implicated as being activated by PDGFR (19). Our data showed that pretreatment with either the PLK4 inhibitor (CenB) or the PLK1 inhibitor (GSK461364) did not significantly alter AA-induced phosphorylation of the MAPK pathway kinases (MEK/ERK, JNK, P38) or AKT/S6K within 40 minutes (Figures 7 and 8). We then dissected which of the PDGFR downstream kinases regulated PLK4 phosphorylation with the use of a panel of their respective inhibitors (Figure 9). Interestingly, the P38 inhibitor markedly reduced AA-stimulated PLK4 phosphorylation, whereas the other inhibitors did not produce a significant effect (Figure 9A). To confirm the specific role of P38 in PLK4 phosphorylation, we performed P38-silencing experiments, and efficient P38 knockdown was indicated by Western blot analysis, whether its total protein or phosphorylated form (Figure 9B). More interestingly, PLK4 phosphorylation (10 minutes after AA stimulation) was substantially reduced in the cells transfected with the P38-specific siRNA compared with the scrambled siRNA control. We also checked PLK1 phosphorylation, which appeared to be reduced generally by the kinase inhibitors, yet no significance resulted after normalization to its total protein (Figure 9C).

### Blocking PDGFR abrogates AA-stimulated PLK4 protein production

**Figure 10:**
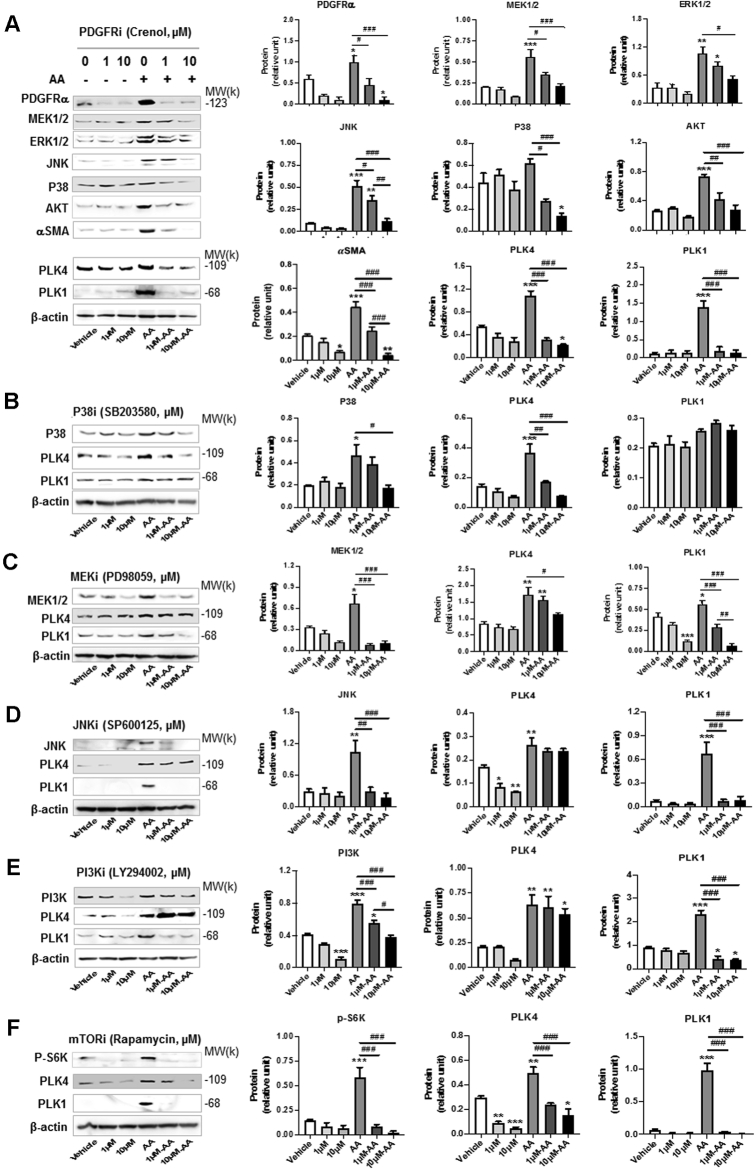
Long-Term Regulation of PLK4 Protein Levels Rat adventitial fibroblast cells were cultured and starved as described in Figure 1. Cells were pretreated for 30 min with vehicle or an inhibitor for **(A)** PDGFR, **(B)** P38, **(C)** MEK/ERK, **(D)** JNK, **(E)** PI3K, or **(F)** mTOR at indicated concentrations and stimulated for 24 h with AA (60 ng/ml) before harvest for Western blot analysis. Quantification: mean ± SEM, n ≥3 experiments, 1-way ANOVA/Bonferroni test: #p < 0.05; ##p < 0.01; ###p < 0.001. ∗p < 0.05; ∗∗p < 0.01; ∗∗∗p < 0.001 compared with the basal control (vehicle without AA, the **first bar in each plot**). Abbreviations as in Figures 1 and 4.

To investigate the long-term regulation of PLK4, we next determined whether PDGFR regulates PLK4 protein levels. As shown in Figure 10A, pretreatment of the cells with the PDGFR blocker crenolanib for 24 h concentration-dependently inhibited PLK4 and PLK1 protein up-regulation that was induced by AA. Serving as a positive control, PDGFR inhibition markedly reduced the protein levels of MEK, ERK, JNK, and P38. Inhibition of mammalian target of rapamycin with rapamycin, which tamps down general protein production, reduced PLK4 and PLK1 proteins, providing another positive control (Figure 10F). In the experiments to dissect the kinases downstream of PDGFR that possibly regulated PLK4 protein expression (Figures 10B to 10E), we observed marked decrease of PLK4 protein after the pretreatment of cells with the inhibitor of P38 but not with that of other kinases. In contrast, PLK1 protein was reduced generally by the kinase inhibitors, suggesting differential regulatory mechanisms for PLK1 and PLK4. Together, these results provide the finding that PDGFRs (most likely PDGFRα, which is specifically activated by AA) stimulates both short-term (5–10 min) PLK4 and PLK1 phosphorylation and a more sustained (24 h) effect seen as increased production of these proteins.

### Pan-BET inhibition blocks AA-stimulated cell–type transition of rat aortic adventitial fibroblasts

**Figure 11:**
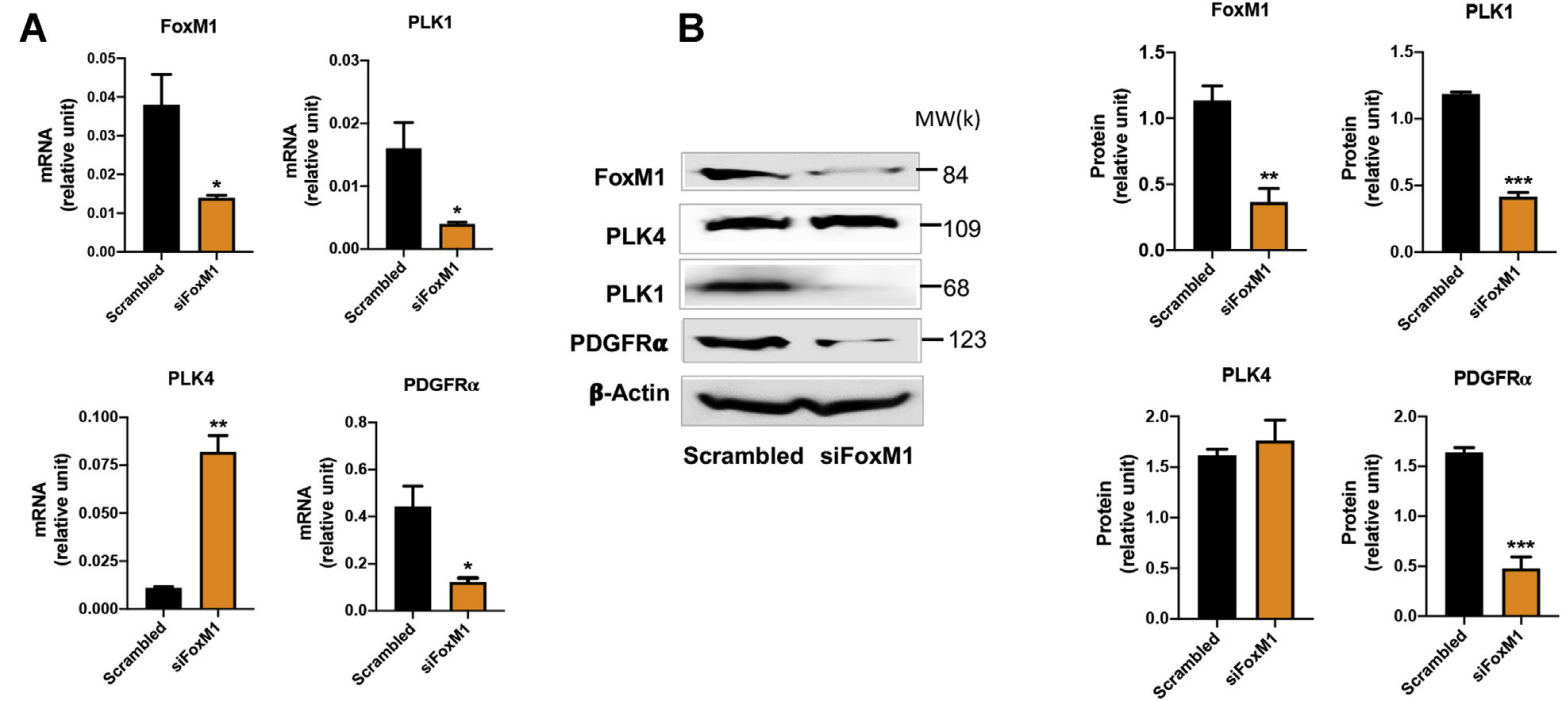
Effects of FoxM1 Silencing on the Transcription of PLK4 and PLK1 Rat adventitial fibroblasts were transfected with scrambled or FoxM1-specific siRNA for 48 hours and then collected for **(A)** qRT-PCR and **(B)** Western blot assays. Quantification: Mean ± SEM, n = 3, Student *t* test: ∗p < 0.05; ∗∗p < 0.01; ∗∗∗p < 0.001. qRT-PCR = quantitative real-time polymerase chain reaction; other abbreviations as in Figures 1 and 4.

We next investigated the transcriptional regulation of PLK4. FoxM1 is a well known transcriptional activator of mitotic regulatory genes, including cyclin B1, Topo2, and Aurora B kinase, as well as PLK1 (6). It was therefore logical to test whether FoxM1 also targets the PLK4 gene. We saw efficient FoxM1 reduction at both the mRNA and the protein level by silencing FoxM1 with an siRNA. This led to substantial reduction of PLK1 mRNA and protein expression (Figure 11), consistent with previous reports (5,6). However, FoxM1 knockdown did not reduce PLK4 mRNA or protein levels.

**Figure 12:**
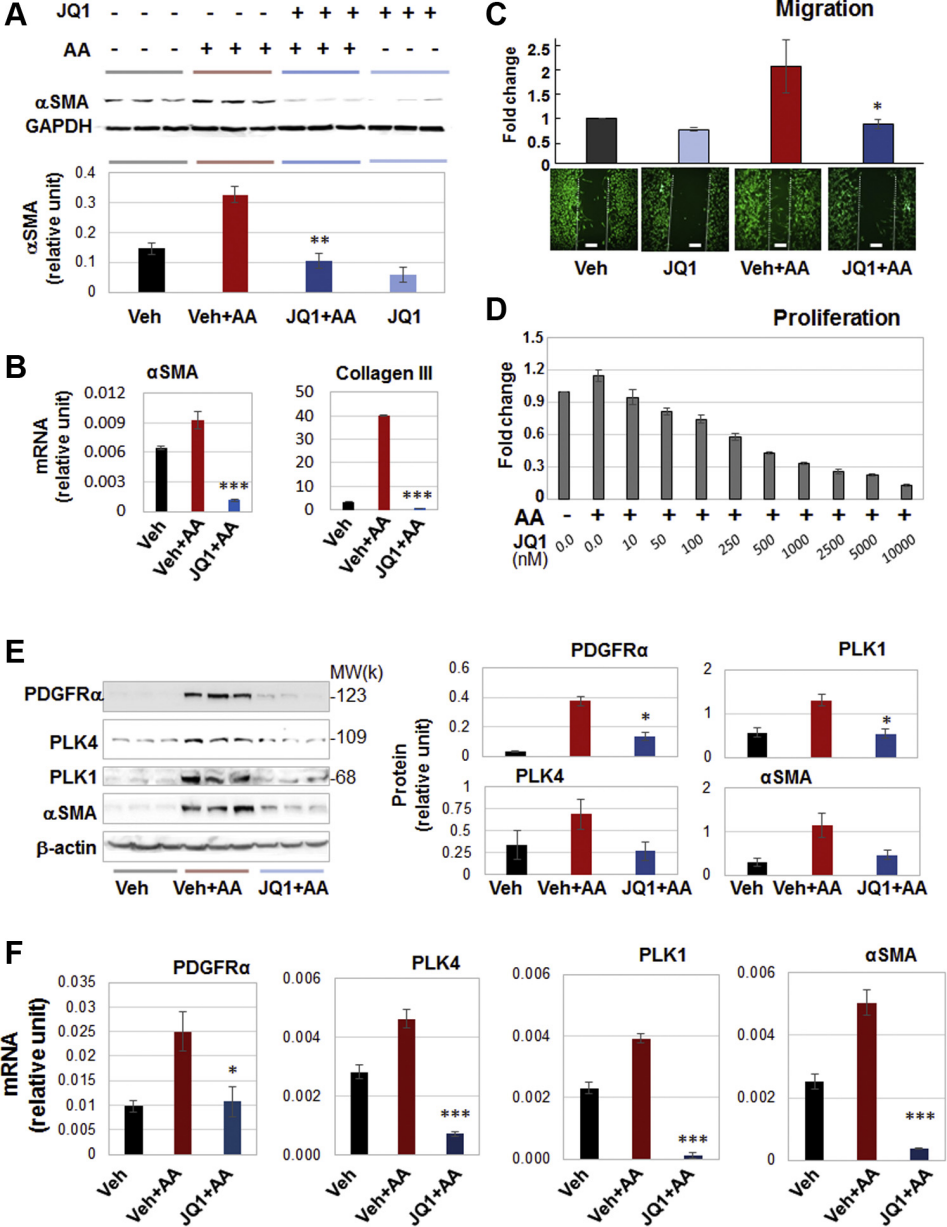
BET Inhibition Blocks AA-Stimulated Fibroblast Cell-Type Transition and Expression of PLK4 and PDGFRα Rat adventitial fibroblasts were cultured and starved as described in Figure 1. Cells were pretreated with vehicle control or JQ1 (1 μmol/l or otherwise specified) overnight before AA stimulation and cell harvest for various assays. **(A, B)** Western blots and qRT-PCR showing JQ1 blockade of AA-stimulated αSMA expression. Densitometry was normalized to GAPDH. **(C)** Migration (scratch assay). Cells were (or were not) stimulated by AA for 48 h without or with pretreatment (1 μmol/l JQ1). Calcein was used to illuminate the cells. **(D)** Proliferation. CellTiter-Glo assay was performed after 72 h AA stimulation of cells without or with pretreatment by increasing concentrations of JQ1. **(E)** Western blots indicating that pretreatment with JQ1 (1 μmol/l, 2 h) abrogated AA-stimulated (24 h) protein production of PDGFRα, PLK4, PLK1, and αSMA. Shown are representative blots from 1 of 2 similar repeat experiments. Densitometry was normalized to β-actin. **(F)** quantitative real-time polymerase chain reaction data indicating that pretreatment with JQ1 (1 μmol/l, 2h) abrogated AA-stimulated (24 h) mRNA expression of PDGFRα, PLK4, PLK1, and αSMA. Quantification: mean ± SD of triplicates; shown is one of two similar experiments. One-way ANOVA/Bonferroni test: ∗p < 0.05; ∗∗p < 0.01; ∗∗∗p < 0.001 compared with the condition of AA + vehicle. BET = bromo/extraterminal domain–containing protein; JQ1, a BET family–selective epigenetic modulator drug; other abbreviations as in Figures 1, 4, and 11.

To further identify key regulatory mechanisms that control PLK4 transcription, we explored the role of bromo/extraterminal domain–containing proteins (BETs). While cell identities are governed by specific transcription programs, recent research discovered that BETs function as master epigenetic regulators, directing transcription programs in a cell-type and environment-dependent manner (12,26, 27, 28). Moreover, recent evidence supports an important role for BETs in activation of fibroblasts (29,30) albeit without data obtained from vascular fibroblasts. As shown in Figures 12A to 12D, pretreatment with JQ1, the first-in-class inhibitor selective for BETs (31) abrogated AA-induced myofibroblastic phenotypes, including αSMA and collagen expression, cell migration, and proliferation, as well as proinflammatory cytokines (Supplemental Figure S4). Apparently, pan-BET inhibition with JQ1 resembles the effect of PLK4 inhibition with CenB (Figure 1). We were therefore motivated to determine the effect of BET inhibition on PLK4 (and PLK1) expression.

### BRD4 predominantly controls the transcription of PLK4 and PDGFRα

Interestingly, pretreatment of the rat fibroblasts with JQ1 averted AA-induced up-regulation of the mRNAs and proteins of PLK4 (and PLK1), a result not previously reported. Moreover, AA-stimulated PDGFRα mRNA/protein expression was also effectively reduced by pretreatment with JQ1 (Figures 12E and 12F).

**Figure 13:**
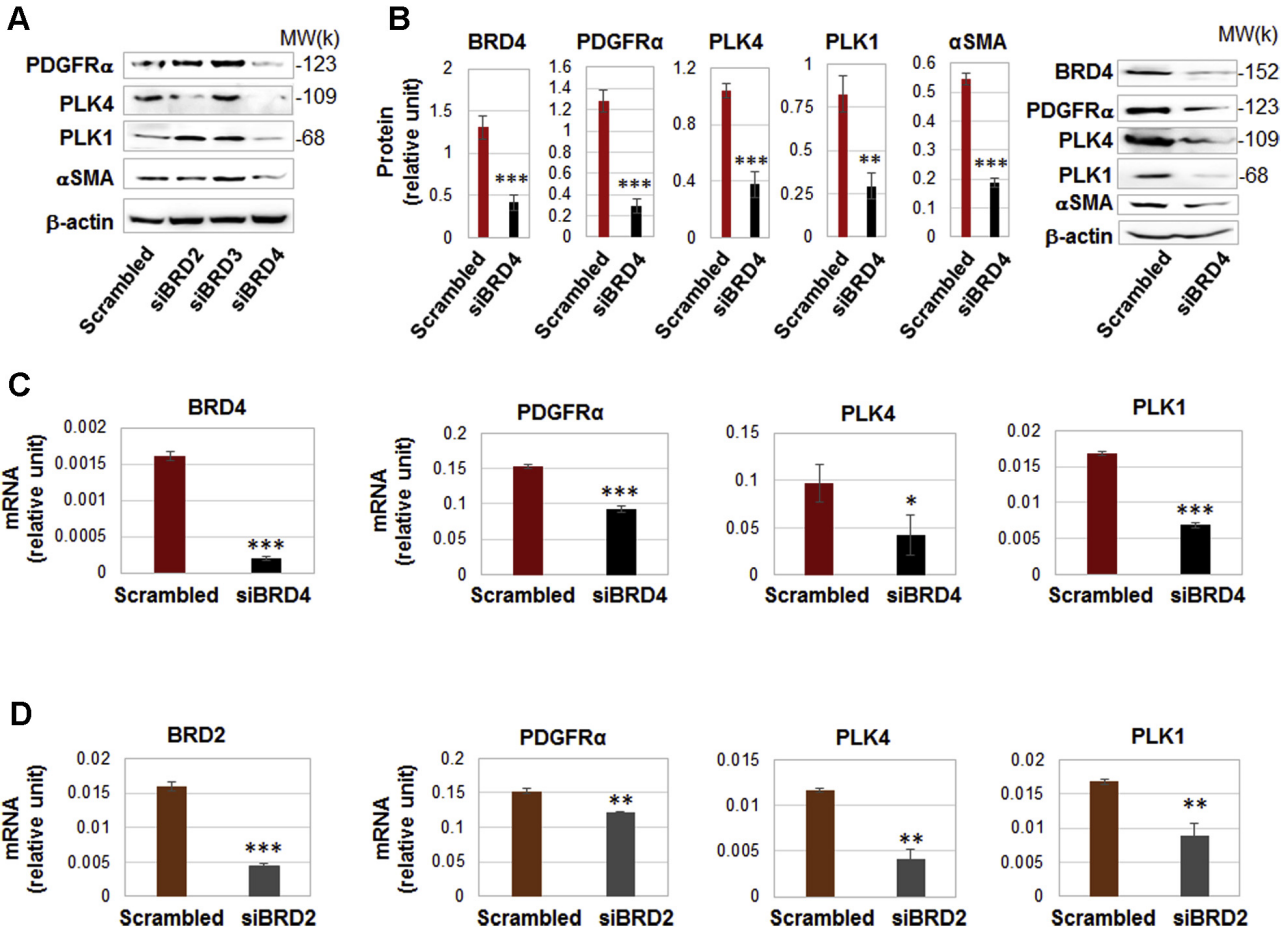
Silencing BRD4 Down-Regulates the Transcription of PLK4 and PDGFRα Rat adventitial fibroblasts were transfected with a scrambled or BRD-specific siRNA for 48 hours and collected for **(A)** Western blot and **(B)** qRT-PCR assays. **(A)** Comparison of the effects of silencing BRD2. BRD3, or BRD4 on PLK4 protein levels. Shown are representative blots from 1 of 2 similar repeated experiments. **(B)** Silencing BRD4 reduces the protein levels of PDGFRα, PLK4, PLK1, and αSMA. Quantification: densitometry normalized to β-actin, mean ± SEM, n = 5 independent experiments, representative blots from one of which are shown on the right. Student *t*-test: ∗∗p < 0.01; ∗∗∗p < 0.001. **(C, D)** Effects of BRD4 or BRD2 silencing on mRNA levels of PDGFRα, PLK4, and PLK1 (qRT-PCR data). Quantification: mean ± SD of triplicates; shown is 1 of 2 similar experiments. Student *t*-test: ∗p < 0.05; ∗∗p < 0.01; ∗∗∗p < 0.001. BRD, bromodomain protein; other abbreviations as in Figures 1, 4, and 11.

The BET family has 4 known members: BRD2, BRD3, BRD4, and BRD-T (testis-restricted and thus irrelevant here). The inhibitor JQ1 binds to all of the BETs (31). To dissect out which BET was responsible for the potent effect of the pan-BET inhibitor JQ1, we performed genetic silencing experiments with the use of siRNAs (Figure 13). Each of the siRNAs effected strong knockdown of its specific target (Supplemental Figure S5). Whereas silencing BRD4 substantially reduced protein levels of PLK4, PLK1, PDGFRα, and αSMA; silencing BRD2 slightly reduced the protein level of PLK4 but not that of PLK1, PDGFRα, or αSMA; and silencing BRD3 did not reduce these proteins (Figure 13A). The robust inhibitory effect of BRD4 silencing on PLK4 and the other 3 proteins is indicated by the quantified data in Figure 13B. Silencing either BRD2 or BRD4 reduced the mRNA levels of PLK4, PLK1, and PDGFRα (though to lesser extents with siBRD2) (Figures 13C and 13D). Given these results, BRD4 appeared to be the predominant BET governing the transcription of the 2 PLKs and PDGFRα.

**Figure 14:**
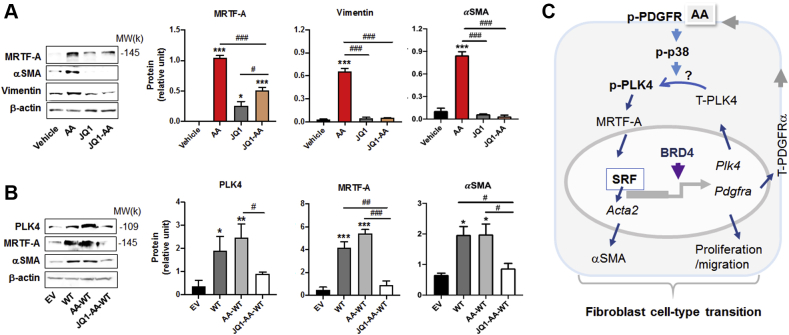
BET Inhibition Blocks MRTF-A and αSMA Expression in the Background of PLK4 Overexpression **(A)** Effect of BET inhibition without PLK4 overexpression. Rat adventitial fibroblast cells were cultured and starved as described in Figure 1. Cells were pretreated with vehicle or JQ1 (1 μmol/l) overnight and then stimulated with AA (60 ng/ml, 24 h) before cell harvest. **(B)** Effect of BET inhibition with PLK4 overexpression. Fibroblast cells (C3H cell line) transduced with lentivirus to express the empty vector control (EV) or PLK4 wild type (WT) were selected with puromycin, starved overnight, pretreated with vehicle or JQ1 (1 μmol/l) overnight, and then stimulated with AA (60 ng/ml, 24 h) before cell harvest. PLK4 was detected by Western blotting against the PLK4 protein. Quantification: mean ± SEM, n = 3 experiments, 1-way ANOVA/Bonferroni test: #p < 0.05; ##p < 0.01; ###p < 0.001. ∗p < 0.05; ∗∗p < 0.01; ∗∗∗p < 0.001 compared with the vehicle or EV control without AA (the **first bar in each plot**). **(C)** Schematic model for regulations up- down-stream PLK4. In an acute phase (within 10 min), AA as extracellular signal initiates activation of the PDGFRα → P38 → PLK4 pathway. PLK4 phosphorylation elevates MRTF-A protein, which in turn activates SRF and αSMA expression. At the transcription level, BRD4 as an epigenetic determinant promotes the transcription of PLK4, as well as PDGFRα, which further enhances PLK4 phosphorylation. The **“?”** mark indicates that our current data cannot distinguish whether P38 is indirectly or directly responsible for PLK4 phosphorylation. Abbreviations as in Figures 1, 2, 4, 12, and 13.

We next explored a possibility as to whether BRD4 acted downstream of PLK4 in regulating fibroblast phenotypic transition. We first saw that inhibiting BRD4 with JQ1 abolished the up-regulation of MRTF-A protein as well as downstream effectors vimentin and αSMA (Figure 14A), consistent with the result presented above (Figure 12). We then elevated PLK4 WT protein levels, and a remarkable increase of MRTF-A and αSMA resulted (Figure 14B). This increase due to PLK4 gain-of-function was completely blocked by JQ1, an effect in accordance with the BRD4 control over PLK4 expression (Figure 13). If BRD4 were downstream or independent of PLK4, with a background of forced overexpression of PLK4, the effect of JQ1 on attenuating MRTF-A should be minor, which was not the case observed here. Based on the above collective results, a diagram of possible upstream and downstream PLK4 regulations, whether its gene expression or its activation (phosphorylation), is presented in Figure 14C. The most likely scenario is that while BRD4 determines PLK4’s gene expression. AA activates the PDGFRα → P38 pathway which leads to PLK4 activation and consequently increased MRTF-A protein and fibroblast transition to a myofibroblast-like state.

### Periadventitial administration of PLK4 inhibitor ameliorates vascular fibrosis in the rat carotid artery injury model

**Figure 15:**
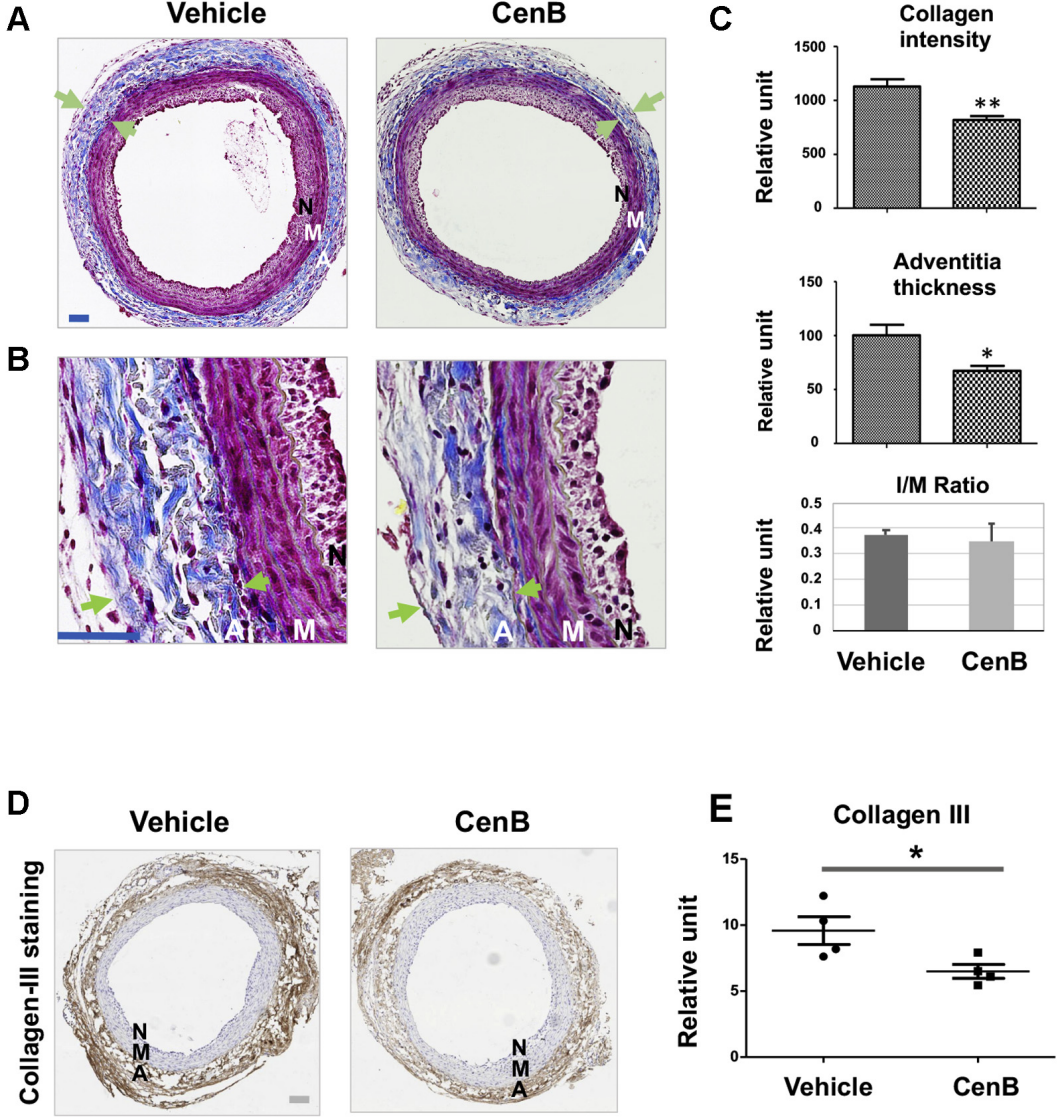
Perivascular Administration of PLK4 Inhibitor Reduces Collagen Content and Thickness of the Adventitia in the Model of Rat Carotid Artery Injury After balloon angioplasty of the rat common carotid artery, vehicle or PLK4 inhibitor (CenB, 100 μg per rat) dissolved in a hydrogel mix was applied around the adventitia of the injured artery. Arteries were harvested at day 7 after injury; cross-sections were used for Masson’s trichrome staining for collagen. **(A)** Representative sections from the arteries treated with vehicle (equal amount of DMSO) or CenB. Collagen is stained **blue**; the adventitia thickness is indicated by 2 **arrows**. The anatomy of the artery wall is labeled as **A** (adventitia), **M** (media), and **N** (neointima). Scale bar: 100 μm. **(B)** Magnified areas of the images in **A**. **(C)** Quantification. Collagen content (staining intensity) and thickness of the adventitia were normalized to the overall vessel size measured as the length of the external elastic lamina (the border between **blue and red layers**). Neointimal hyperplasia was measured as the intima/media area ratio (I/M). Mean ± SEM, n = 5 animals per group. Student *t*-test: ∗p < 0.05; ∗∗p < 0.01. **(D)** Representative immunohistologic images for collagen III. Scale bar: 100 μm. **(E)** Quantification. The staining intensity (measured with the use of ImageJ above a threshold) was normalized to the perimeter of the external elastic lamina. The data from different sections were pooled to generate the mean for each animal. The means from all animals in each group were then averaged, and the final mean ± SEM was calculated. Student *t*-test: ∗p < 0.05; n = 4 animals per group indicated by the 4 data points in the plot. Abbreviations as in Figure 1.

**Figure 16:**
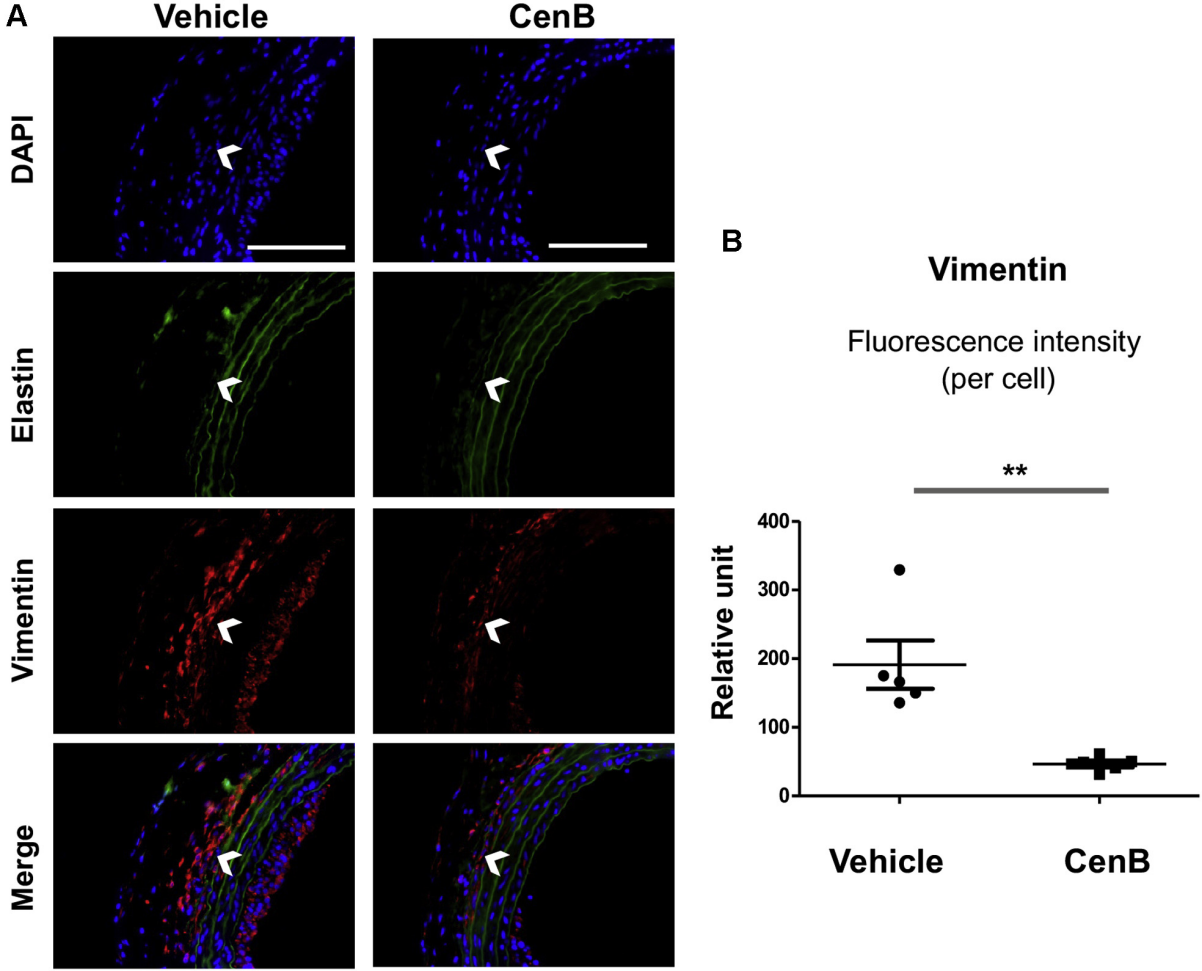
Perivascular Administration of PLK4 Inhibitor Reduces Vimentin in the Adventitia of Injured Arteries **(A)** Representative immunofluorescence images for vimentin. Elastin autofluorescence was used to distinguish the external elastic lamina (indicated by **open arrowheads**), the border between adventitia and media. Scale bar: 100 μm. **(B)** Quantification. The immunofluorescence intensity in the adventitial layer (measured with the use of ImageJ above a threshold) was normalized to the number of 6-diamino-2-phenylindole (DAPI)–stained corresponding adventitial cells in each image field. The data from different sections were pooled to generate the mean for each animal. The means from all animals in each group were then averaged, and the final mean ± SEM was calculated. Student *t*-test: ∗∗p < 0.01; n = 5 animals per group. Abbreviations as in Figure 1.

**Figure 17:**
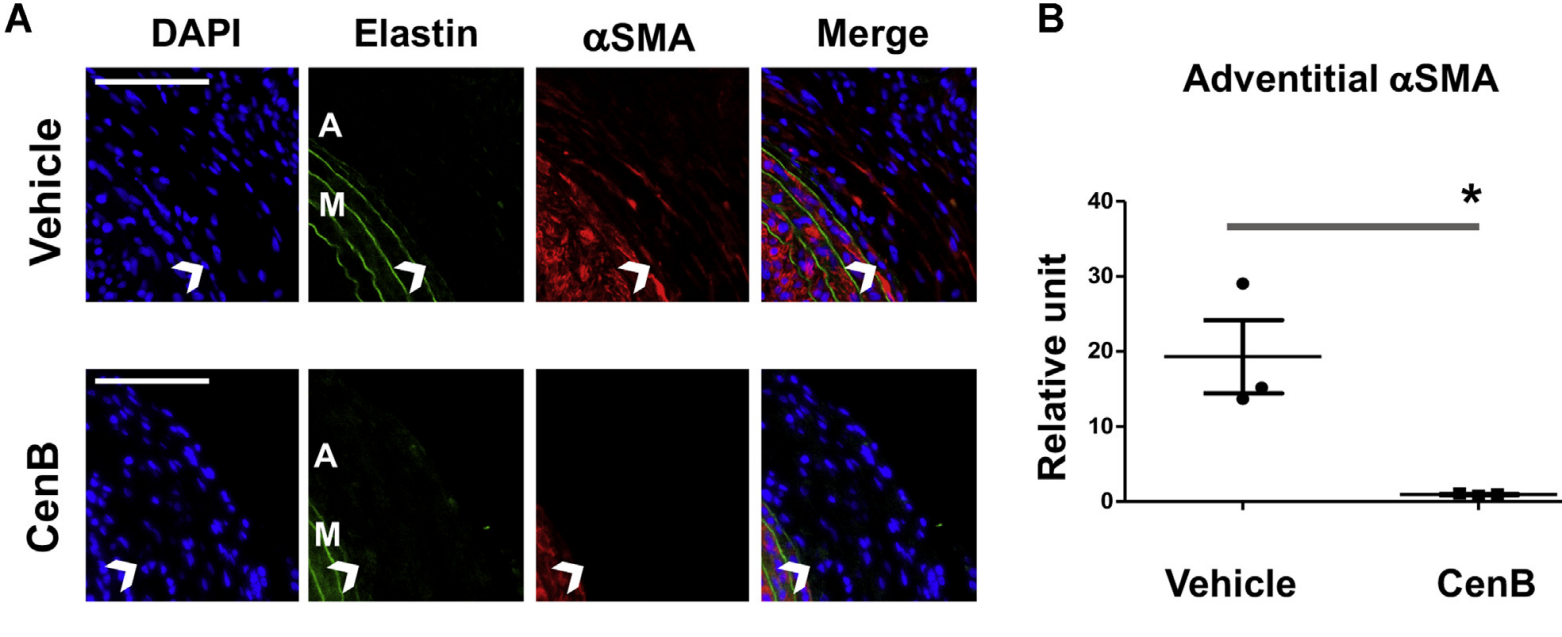
Perivascular Administration of PLK4 Inhibitor Reduces αSMA in the Adventitia of Injured Arteries A. Representative immunofluorescence images for αSMA. Elastin autofluorescence was used to distinguish the external elastic lamina (indicated by **open arrowheads**). The strong αSMA signal in the media layer, where smooth muscle cells (high αSMA expression) reside, presents a positive control validating αSMA immunostaining. Scale bar: 100 μm. **(B)** Quantification. The immunofluorescence intensity in the adventitial layer (measured with the use of ImageJ above a threshold) was normalized to the number of DAPI-stained corresponding adventitial cells in each image field. The data from different sections were pooled to generate the mean for each animal. The means from all animals in each group were then averaged to produce the final mean ± SEM (n = 3 animals per group). Student *t*-test: ∗p < 0.05.

After identifying the role for PLK4 in fibroblast cell-type transition and the regulators of its phosphorylation and expression, we then determined whether PLK4 inhibition reduces fibrosis in vivo. We used a well established rat arterial injury model in which injury-induced fibrosis manifests as high collagen content in the adventitia. Balloon angioplasty was performed in rat common carotid arteries, and the PLK4 inhibitor (CenB) or vehicle control was administered in thermosensitive hydrogel distributed around the injured artery, following our previously reported method (12). Cross-sections of injured arteries collected at day 7 were stained with the Masson’s trichrome method (14) (Figures 15A and 15B). Quantified data indicated that the collagen content was significantly reduced in CenB-treated arteries compared with vehicle control, as also confirmed by the immunohistochemistry of collagen III (Figures 15D and 15E). Consistently, the adventitia thickness also significantly decreased after CenB treatment (Figure 15C). Aside from collagen content measurement, immunofluorescence staining was performed to determine the levels of vimentin and αSMA, important markers of fibroblast activation. Although vimentin expression mostly occurred in the artery adventitial layer where fibroblast (or its activated form) primarily resides, periadventitial treatment with CenB reduced vimentin protein levels in the artery wall by >70% (Figure 16). Marked decrease of αSMA staining in the adventitial layer was also observed as a result of CenB treatment (Figure 17). The strong αSMA signal in the medial layer of smooth muscle cells presents a positive control validating αSMA immunostaining. Thus, these in vivo results concurred with the CenB effect on fibroblast cells in vitro (Figure 1). No significant change was observed in neointimal hyperplasia (measured as the neointima/media area ratio) (12). An interesting question for future investigation is whether CenB treatment reduces neointima at day 14, a time point when the neointima thickness is maximal. In aggregate, the results presented herein indicate that in this model of rat carotid artery injury, periadventitial application of the PLK4 inhibitor CenB ameliorated vascular fibrosis.

## Discussion

The major findings of this study are: 1) Though previously known as centriole-specific, PLK4 positively regulated MRTF-A protein levels, SRF nuclear activity, and the target gene αSMA’s expression; 2) upon AA stimulation, PLK4 phosphorylation was activated by PDGFR and downstream kinase P38; and 3) the transcription of PLK4 was predominantly governed by epigenetic reader BRD4. Thus, our results unravel a noncanonical role for PLK4 in SRF activation and fibroblast cell-type transition, and further uncover a BRD4/PDGFR–dominated mechanism underlying PLK4’s expression/kinase activation. Importantly, PLK4 inhibition was effective in attenuating vascular fibrosis in an arterial injury rat model.

The PLK4’s canonical role in centriole duplication has been recently reviewed (32). Briefly, PLK4 phosphorylates and complexes with STIL, which in turn recruits SAS6 (spindle assembly abnormal protein 6 homolog), forming the core module of centriole genesis. However, little has been known about noncanonical PLK4 functions (3,33). Few of PLK4’s substrates beyond centriolar proteins have been identified (33). Thus, PLK4 is much less understood than PLK1, the best studied PLK member, which mediates multiple mitotic processes (1,32).

Given that PLK4 is a centriole-associated factor, it was not surprising to learn that PLK4 is proproliferative (1) in vascular fibroblasts, as observed here, and in cancer progression, as previously reported (34,35). PLK4 was also recently linked to cancer cell migration and invasion (33), consistent with our result that PLK4 inhibition mitigates vascular fibroblast migration. On the other hand, cell-type transition is a process beyond cell proliferation and migration; it involves remodeling of signaling and transcription programs (8) and thereby results in altered cell type with multiple phenotypic changes. In this regard, it is an unanticipated finding that PLK4, known as a centriole-specific factor, assumes a noncanonical role enabling profound cell-type transformation.

Elevated expression of αSMA, which is governed by the master transcription factor SRF, endows cells with a contractile function (8). Our data showed that while PLK4 inhibitor profoundly reduced SRF’s activity, PLK4 loss- and gain-of-function substantially reduced and elevated αSMA levels, respectively. This was somewhat unexpected, given that PLK4 acts in the cytosol for its centriole-associated function whereas SRF is a chromatin-associated nuclear protein. Interestingly, this paradox could be rationalized by our novel observation that PLK4 positively regulates protein levels (or stability) of MRTF-A. This cofactor of SRF is a cytosol-nucleus shuttling protein and may thereby convey PLK4’s function to SRF activation.

This noncanonical function of PLK4 in SRF activation is intriguing, especially considering that the SRF/αSMA transcriptional remodeling is both phenotypically and mechanistically distinct from cell proliferation and migration. In a recent report in cancer research, PLK4 was found to play a role in cytoskeleton reorganization and lamellipodia formation in HeLa cells (33), and the authors used the result to explain the PLK4 function in cancer cell migration. Similarly, an earlier study showed that PLK4 expression enhanced the polarity, spreading, and invasion of colon cancer cells (36). However, whether PLK4 played a role in αSMA transcription was not examined in either of these studies. Another line of evidence is from a very recent report that PLK4 up-regulation promoted epithelial cell state transition in neuroblastoma (37). Although that report showed that PLK4 enhanced vimentin expression, there were no data on αSMA transcription or SRF activity. In addition, a new report demonstrated that PLK1 positively regulated angiotensin II activation of Ras homolog A and actomyosin dynamics in murine vascular smooth muscle cells (11). However, caution must be taken with the analogy of PLK4 with PLK1. Published evidence and our own results indicate that although in the same family, PLK4 and PLK1 may functionally vary, especially in different cell types or states. Nevertheless, the positive regulation of MRTF-A/SRF by PLK4 represents a distinct mechanism that may or may not cross-talk with those reported previously. In this light, a PLK4 noncanonical role in regulating MRTF-A and SRF activities warrants future studies for further elaboration.

Given PLK4’s critical role in centrosome biogenesis and its potential noncanonical functions, this kinase is expected to be intricately regulated (38), yet relatively little is known about the regulation of PLK4 in mammalian systems (20,39). We found that activation of PLK4 (phosphorylation at T170) (20,38) by AA treatment was blocked by PDGFR inhibition. Because AA specifically activates the PDGFR αα homodimer (10), we inferred that it was primarily the activation of PDGFRα that led to PLK4 phosphorylation. We reasoned that the PDGFRα activation of PLK4 was indirect because PDGFRα is a receptor tyrosine kinase (10) with no known threonine kinase activity. Downstream of PDGFR, P38 appeared to be an activator of PLK4, as evidenced by experiments using either a P38 inhibitor or siRNA. In contrast, PLK4 inhibition did not significantly alter PDGFRα or P38 phosphorylation, placing PLK4 downstream of the PDGFR/P38 signaling. Our results do not reveal whether PLK4 is a direct substrate of the P38 kinase. This question could be addressed in future studies using both purified proteins. Although P38-mediated PLK4 activation has not been previously reported, evidence consistent with this finding comes from a recent in vivo study where P38 proved to be a critical myofibroblastic activator (40). Additional evidence from our data is that inhibiting either PDGFR or P38 averted AA-induced PLK4 protein up-regulation. In contrast, pretreatment with inhibitors of other PDGFR downstream pathways (e.g., MEK/ERK and JNK) did not produce an obvious effect on PLK4 protein levels. Taken together, our results have profiled a PDGFR → P38 → PLK4 → MRTF-A → SRF signal transduction pathway (Figure 14C).

In pursuit of the molecular mechanism underlying transcriptional regulation of PLK4, we investigated representatives of 2 different categories of regulators, namely, that known to regulate the members of the PLK family (FoxM1) and that known to regulate myofibroblastic activation (BETs). Interestingly, our data showed that silencing transcription factor FoxM1, a known regulator of PLK1, did not repress PLK4 expression, at either mRNA or protein level. Instead, we found that BET family member BRD4 played a predominant role in governing PLK4 gene transcription. This is consistent with previous reports indicating a positive role of BETs in fibroblast activation (29,30). Those studies showed that BET family inhibitors mitigated myofibroblast transdifferentiation and fibrosis in vital organs such as the lung, liver, pancreas, and heart (28, 29, 30,41). Our study differs from those reports by addressing 2 important questions: 1) Do any of the BETs regulate PDGFRs? and 2) do any of the BETs regulate PLK4 or PLK1? Given our finding that silencing BRD4 effectively reduced mRNA and protein levels of PDGFRα, PLK4, and αSMA, BRD4 appears to play a master role in governing this entire PLK4 pathway. Indeed, in recent reports BRD4 emerged as a master epigenetic regulator in a variety of cell-state/type transitions (12,27, 28, 29). While a cell-type/state transition often results from environmental perturbations, environmental cues (e.g., PDGF-AA used here) and the resultant transcription reprogramming represent 2 ends of this event, and an epigenetic regulator such as BRD4 functions at their interface. On stimulation, BRD4 acts as a key organizer of trans- and cis-elements (e.g., superenhancers) and the core transcription machinery, and (re)localizes them to specific sets of genes to activate their transcription (42). The bromodomains of BETs “usher” this transcription assembly to target genes by binding to bookmarked (acetylated) chromatin loci. This BRD4-directed mechanism has been recently recognized as critical in orchestrating cell-state transitions in various pathobiological contexts (12,26, 27, 28, 29). As indicated by our data, BRD2 also participated in regulating the PLK4 pathway but played a lesser role (compared with BRD4). The mechanistic difference between the effects of BRD4 and BRD2 on PLK4 awaits further investigation.

## Conclusions

We have made an unexpected finding that PLK4, a kinase traditionally known as a centriole duplication factor, regulates cell-type transitions of vascular fibroblasts under PDGF-AA stimulation. The significance of our study is 3-fold. First, PLK4’s positive regulation of MRTF-A protein and SRF activation is a novel function. Second, while PLK4 is a central player in the PDGFRα → P38 → PLK4 → SRF pathway that prompts PDGF-induced αSMA expression, BRD4 governs the entire pathway as an epigenetic determinant of gene expression. In this context, PLK4 appears to be a signaling effector in sensing and transmitting environmental cues. Third, consistent with the in vitro role of PLK4 in a myofibroblast-like cell-type transition, PLK4 inhibition mitigates vascular fibrosis in vivo, implicating a new antifibrotic strategy.

Perspectives **COMPETENCY IN MEDICAL KNOWLEDGE:** Fibrosis can occur in essentially every organ, leading to a wide spectrum of pathologies, including cardiovascular diseases. As such, its etiology is extremely complex, posing tremendous challenges for developing effective treatments. It is thus imperative to identify potential intervention targets through context-dependent mechanistic studies. Our study in the vascular setting implicates PLK4 as an unconventional antifibrotic target. Further translational research is warranted, especially as PLK4-selective inhibitors have recently been developed and applied in clinical tests for cancer therapy.

**TRANSLATIONAL OUTLOOK 1:** To examine the robustness of a PLK4-targeted antifibrotic strategy, other distinct preclinical models of vascular fibrosis should be used.

**TRANSLATIONAL OUTLOOK 2:** To better understand the benefits and unwanted effects of PLK4 inhibition in treating vascular fibrosis, future studies should include other vital vascular cell types, namely, smooth muscle cells and endothelial cells, as well as nonvascular cells such as macrophages.

## Funding Support and Author Disclosures

This work was supported by National Institutes of Health grants R01 HL133665 (to Dr. Guo) and R01HL-143469 (to Drs. Kent and Guo) and American Heart Association predoctoral award 17PRE33670865 (to Dr. Zhang). All other authors have reported that they have no relationships relevant to the contents of this paper to disclose.
