# Supplementary material for: A Role for Polo-Like Kinase 4 in Vascular Fibroblast Cell-Type Transition
Source: JACC Basic Transl Sci. 2021 Mar 22;6(3):257–83. doi: 10.1016/j.jacbts.2020.12.015 (PMC7987547; doi:10.1016/j.jacbts.2020.12.015)
Supplement: Supplemental Tables 1–6 and Supplemental Figures 1–5 [file mmc1.docx]

SUPPLEMENTAL MATERIAL

**Supplemental Figure S1 αSMA and Vimentin Expression in Rat Adventitial Fibroblasts at Different Passages**

**
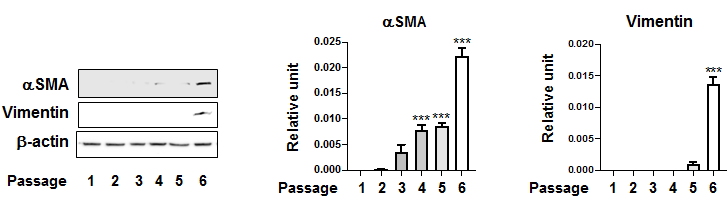
**

Rat primary aortic adventitial fibroblasts were isolated and cultured in the complete medium to expand to various passages before harvest for Western blot analysis. Quantification: mean ± SEM, n =3 experiments, 1-way ANOVA/Bonferroni test: ***p < 0.001 compared with passage 1 (data value is too low to appear as a bar in the plot). αSMA = α-smooth muscle actin; ANOVA = ANOVA.

**Supplemental Figure S2 PLK4 Inhibition Attenuates TGFβ1-Stimulated Vimentin Expression in Rat Adventitial Fibroblasts**

**
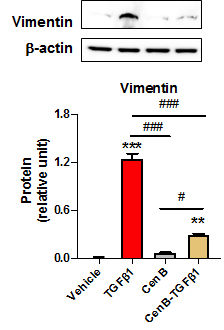
**

Rat primary adventitial fibroblasts were cultured in the complete medium, starved in the basal medium overnight, and pretreated for 2 hours with vehicle (equal amount of DMSO) or CenB (1 µmol/L), followed by stimulation with 20 ng/mL transforming growth factor (TGF) β1. Cells were harvested at 24 hours after stimulation for Western blot analysis. Quantification: mean ± SEM, n = 3 experiments, 1-way ANOVA/Bonferroni test: #p < 0.05; ###p < 0.001; **p < 0.01; ***p < 0.001 compared with control (vehicle, no TGFβ1). CenB = centrinone-B; DMSO = dimethylsulfoxide; PLK = Polo-like kinase; other abbreviations as in Figure S1.

**Supplemental Figure S3 MRTF-A and αSMA Protein Levels in Fibroblast Cell Cytosol and Nucleus**


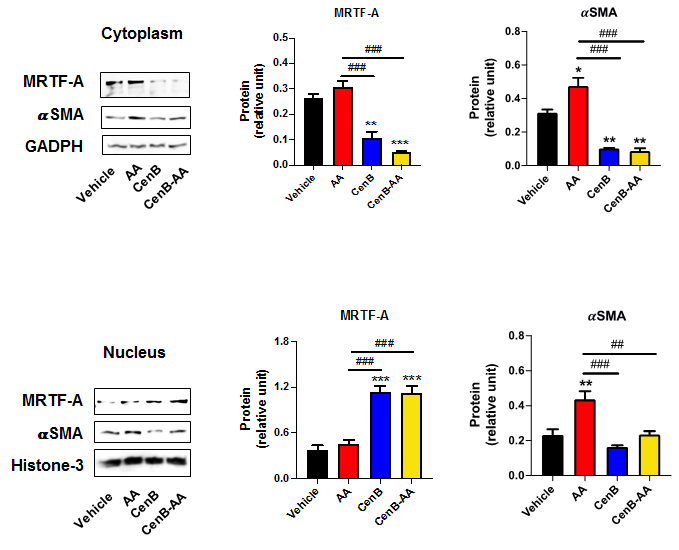


Rat primary adventitial fibroblasts were cultured in the complete medium, starved in the basal medium overnight (see Methods), and pretreated for 2 hours with vehicle (equal amount of DMSO) or the PLK4-selective inhibitor CenB (1 μmol/L), followed by stimulation with 60 ng/mL PDGF-AA for 24 hours. Cells were then harvested for separation of nuclei from cytosol and Western blot analysis. The nucleus and cytosol extracts were prepared with NE-PER Nuclear and Cytoplasmic Extraction Reagents (cat. 78835; Invitrogen) according to the manufacturer’s instruction. Briefly, cell pellets were washed with cold phosphate-buffered saline solution and resuspended in 200 μL cytoplasmic extraction reagent I by vortexing. The suspension was incubated on ice for 10 minutes, followed by the addition of 11 μL cytoplasmic extraction II and vortexing, and then centrifuged for 10 minutes at 16,000*g*. The supernatant fraction (cytoplasmic extract) was transferred to a prechilled tube and used for the experiment. The pellet fraction was resuspended in 100 μL of nuclear extraction reagent by vortexing at least 4 times each for 15 seconds, incubated on ice for 10 minutes after each vortexing, and then centrifuged for 10 minutes at 16,000*g*. The supernate (nuclear extract) was kept on ice until use for experiments. MRTF-A = myocardin-related transcription factor A; PDGF = platelet-derived growth factor; other abbreviations as in Figures S1 and S2.

**Supplemental Figure S4. JQ1 Inhibits PDGF-AA–Stimulated Inflammation and Collagen Expression**


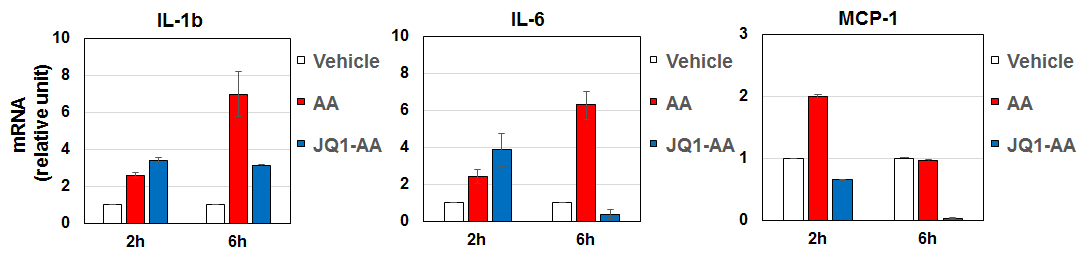


The quantitative real-time polymerase chain reaction assay was performed in the same experiments as in Figure 12. Briefly, rat primary adventitial fibroblasts were cultured, starved, pretreated with JQ1 before PDGF-AA stimulation. Quantification: mean ± SD, n = 3, 1 of 2 similar repeat experiments. IL = interleukin; JQ1 = a BET family–selective epigenetic modulator drug.

**Supplemental Figure S5. siRNA Knockdown Efficiency for BRD2, BRD3, and BRD4**

**
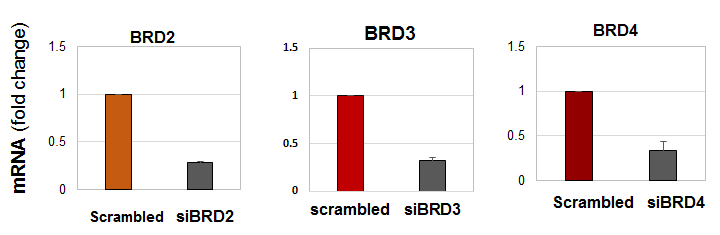
**

The quantitative real-time polymerase chain reaction assay was performed in the same experiments as in Figure 13. Quantification: mean ± SD, n = 3, 1 of 2 similar repeat experiments. BRD = bromodomain protein.

**Supplemental Table S1. BET and Kinase Inhibitors**

| **Inhibitor** | **Target protein** | **Source company** | **Catalog no.** | **Stock solution concentration** |
| --- | --- | --- | --- | --- |
| JQ1(+) | Bromodomain inhibitor | ApexBio | A1910 | 10 mmol/L |
| GSK461364 | PLK1 | ApexBio | A8441 | 10 mmol/L |
| Centrinone-B | PLK4 | Tocris | 5690 | 10 mmol/L |
| Crenolanib | PDGFR | MedChenExpress | HY-13223 | 10 mmol/L |
| Tubastatin A | HDAC6 | Selleckchem | 1252003-15-8 | 10 mmol/L |
| SB230580 | P38 | Calbiochem | 559389 | 10 mmol/L |
| SP600125 | JNK | Calbiochem | 420119 | 20 mmol/L |
| LY294002 | PI3K | Calbiochem | 440202 | 20 mmol/L |
| Rapamycin | mTOR | Calbiochem | 553210 | 10 mmol/L |
| PD98059 | MEK1/2 | Calbiochem | 513000 | 25 mmol/L |

**Supplemental Table S2. Antibodies for Western blotting**

| **Antibody** | **Company** | **Catalog no.** | **Dilution** |
| --- | --- | --- | --- |
| αSMA rabbit monoclonal | Abcam | ab32575 | 1:1,000 |
| β-Actin mouse monoclonal | Abcam | Ab6276 | 1:5,000 |
| Anti–γ-actin rabbit polyclonal | Proteintech | 11227-1-AP | 1:1,000 |
| GADPH rabbit monoclonal | CST | 2118 | 1:1,000 |
| Vimentin (H-84) rabbit polyclonal | Santa Cruz | sc-5565 | 1:1,000 |
| MKL1/MRTF-A rabbit polyclonal | CST | 14760 | 1:1,000 |
| PDGFRα rabbit polyclonal | CST | 3164 | 1:1,000 |
| p-PDGFRα | Abcam |  | 1:1,000 |
| p-MEK1/2 (S217/221) | CST | 9154 | 1:1,000 |
| MEK1/2(D1A5) rabbit monoclonal | CST | 8727 | 1:1,000 |
| p-P44/42 MAPK (ERK1/2)(T202/Y204) | CST | 4370 | 1:1,000 |
| P44/42 MAPK (ERK1/2) | CST | 9102 | 1:1,000 |
| p-SAPK/JNK (T183/Y185) rabbit monoclonal | CST | 4668 | 1:1,000 |
| SAPK/JNK rabbit | CST | 9252 | 1:1,000 |
| p-P38 MAPK(T180/Y182) rabbit | CST | 9211 | 1:1,000 |
| P38 MAPK rabbit | CST | 9212 | 1:1,000 |
| p-AKT(Ser473) rabbit | CST | 9271 | 1:1,000 |
| AKT pan (C6E7) rabbit monoclonal | CST | 4691 | 1:1,000 |
| p-S6 ribosomal protein (S235/236) rabbit | CST | 2211 | 1:1,000 |
| p-PLK1T210 | Abcam | ab73018 | 1:1,000 |
| PLK1 rabbit polyclonal | Proteintech | 10305-1-AP | 1:1,000 |
| p-PLK4T170 | KINEXUS | AB-PK780 | 1:500 |
| PLK4 rabbit polyclonal | Proteintech | 12952-1-AP | 1:1,000 |
| BRD2 rabbit polyclonal | Proteintech | 22236-1-AP | 1:1,000 |
| BRD3 rabbit polyclonal | Proteintech | 11859-1-AP | 1:1,000 |
| Anti-BRD4 [EPR5150(2)] | Abcam | Ab128874 | 1:1,000 |
| FOXM1 rabbit polyclonal | Proteintech | 13147-1-AP | 1:1,000 |
| Goat antimouse (H+L) HRP-conjugated IgG | Bio-Rad | 170-6516 | 1:3,000 |
| Goat antirabbit (H+L) HRP-conjugated IgG | Bio-Rad | 170-6515 | 1:3,000 |

**Supplemental Table S3. Small interfering RNAs (Rat Sequence)**

| **Target gene** | **Sense** | **Antisense** |
| --- | --- | --- |
| BRD2 | GCUUGAACGAUACGUUUUA | UAAAACGUAUCGUUCAAGC |
| BRD3 | AGGAAACCAUUGUCAACAATT | UUGUUGACAAUGGUUUCCUCT |
| BRD4 | GCAUCAACUUCUCCGCAGATT | UCUGCGGAGAAGUUGAUGCTT |
| PLK4 | GAUCACCGUUUAUUACCCATT | UGGGUAAUAAACGGUGAUCGT |
| FoxM1 | GUCCAUUAAGGAAGAAGUATT | UACUUCUUCCUUAAUGGACTG |
| P38 | GCUUACCGAUGACCACGUUTT | AACGUGGUCAUCGGUAAGCTT |

**Supplemental Table S4. Primers for Quantitative Real-Time Polymerase Chain Reaction (Rat Sequence)**

| **Target gene** | **Forward** | **Reverse** |
| --- | --- | --- |
| αSMA | CCAGGGAGTGATGGTTG | TCTATCGGATACTTCAGGGT |
| Collagen-III | CCACCCTGAACTCAAGAGC | ACCAGCATCTGTCCACCAG |
| BRD2 | CTTCGCTGTTGTATGAGGG | GTTGGTTTGTTACTCGTCCTG |
| BRD4 | CTCAGCAAGTCATCCAGCATC | TCAGCCCTGCCCTTTACC |
| GAPDH | GACATGCCGCCTGGAGAAAC | AGCCCAGGATGCCCTTTAGT |
| FoxM1 | CCTGGTGTTACAGCCCTCG | GGACTCGCTTGCTATGACG |
| PDGFRα | GCTTGGCAAAGAACGAC | TGACAACCAGGACAATGAG |
| PLK4 | CCTGGTATTAGAAATGTGCC | GGTGTAGTATGCCGTGAG |
| PLK1 | TACCTGCCTCACCATCCC | CCTCATTTGTCTCCCGAACC |

**Supplemental Table S5. Primers for PLK4 Mutant Constructs**

| **Primer name** | **Sequence (5′–3′)** |
| --- | --- |
| PLK4 wild type | |
| Forward | GTGGATCCAATGGATTACAAGGATGACGACGATAAGGATTACAAGGATGACGACGATAAGGCGGCGTGCATCGGGGAGAGG |
| Reverse | CGAATTCCATTACTGAAAATTAGGAGTTGGATTAG |
| T170A mutant | |
| Forward | GAAAAGCACTATGCACTCTGTGGGACTC |
| Reverse | GAGTCCCACAGAGTGCATAGTGCTTTTC |
| T170T174A mutant | |
| Forward | GCACTATGCACTCTGTGGGGCTCCT |
| Reverse | AGGAGCCCCACAGAGTGCATAGTGC |
| T170AT174AS179A mutant | |
| Forward | CTATGCACTCTGTGGGGCTCCTAATTATATTGCACCAG |
| Reverse | CTGGTGCAATATAATTAGGAGCCCCACAGAGTGCATAG |
| T138A mutant | |
| Forward | GCACCGGGACCTCGCACTCTCTAACATC |
| Reverse | GATGTTAGAGAGTGCGAGGTCCCGGTGC |
| S305A mutant | |
| Forward | CAGTTTGAGTGGCGCCCTACTTGACAGAAG |
| Reverse | CTTCTGTCAAGTAGGGCGCCACTCAAACTG |

**Supplemental Table S6. Antibodies for Immunostaining**

| **Antibody** | **Company** | **Catalog no.** | **Dilution** |
| --- | --- | --- | --- |
| Vimentin | Santa Cruz | sc-373717 | 1:100 |
| αSMA | Abcam | ab5694 | 1:100 |
| Collagen III | Novus Biologicals | NBP105119 | 1:600 |
